# Organization of biogeochemical nitrogen pathways with switch-like adjustment in fluctuating soil redox conditions

**DOI:** 10.1098/rsos.160768

**Published:** 2017-01-11

**Authors:** Sanjay Lamba, Soumen Bera, Mubasher Rashid, Alexander B. Medvinsky, Gui-Quan Sun, Claudia Acquisti, Amit Chakraborty, Bai-Lian Li

**Affiliations:** 1 School of Mathematics, Statistics and Computational Sciences, Central University of Rajasthan, Bandarsindri, Ajmer, India; 2 Institute of Theoretical and Experimental Biophysics, Pushchino 142290, Russia; 3 Department of Mathematics, Shanxi University, Taiyuan, People's Republic of China; 4 Institute for Evolution and Biodiversity, WWU Muenster, Germany; 5 Ecological Complexity and Modeling Laboratory, University of California, Riverside, CA 92521-0124, USA

**Keywords:** biochemical network, nitrogen cycle, self-regulation, ecosystems, biogeochemistry

## Abstract

Nitrogen is cycled throughout ecosystems by a suite of biogeochemical processes. The high complexity of the nitrogen cycle resides in an intricate interplay between reversible biochemical pathways alternatively and specifically activated in response to diverse environmental cues. Despite aggressive research, how the fundamental nitrogen biochemical processes are assembled and maintained in fluctuating soil redox conditions remains elusive. Here, we address this question using a kinetic modelling approach coupled with dynamical systems theory and microbial genomics. We show that alternative biochemical pathways play a key role in keeping nitrogen conversion and conservation properties invariant in fluctuating environments. Our results indicate that the biochemical network holds inherent adaptive capacity to stabilize ammonium and nitrate availability, and that the bistability in the formation of ammonium is linked to the transient upregulation of the *amo-hao* mediated nitrification pathway. The bistability is maintained by a pair of complementary subsystems acting as either source or sink type systems in response to soil redox fluctuations. It is further shown how elevated anthropogenic pressure has the potential to break down the stability of the system, altering substantially ammonium and nitrate availability in the soil, with dramatic effects on biodiversity.

## 1. Introduction

Nitrogen (N), an essential building block of life supporting macromolecules, is cycled throughout ecosystems by a suite of biogeochemical processes that involve a variety of interdependent biochemical reactions catalysed by microbe-excreted enzymes [1,2]. The core of the N-biochemical network involves four types of reduction (nitrogen fixation, assimilatory nitrate reduction, dissimilatory nitrate reduction to ammonia (DNRA) and denitrification) and two oxidation pathways (nitrification and anaerobic ammonia oxidation). Each pathway involves several biochemical reactions and associated enzymes, leading to the transformation of the oxidation state that ranges from +5 in nitrate to −3 in ammonia (table 1) [3,4]. These biogeochemical processes are particularly susceptible to environmental redox fluctuations. The soil redox state acts as a master switch between alternative pathways with fundamentally different kinetics [5]. In fluctuating redox conditions, soils cycle between oxic and anoxic states leading to activation or inactivation of biochemical reactions which in turn alter the nature and rate of the associated N-transformations [6]. While oxic conditions favour nitrification, anoxic conditions facilitate denitrification and DNRA. This is a critically important aspect to consider in order to deepen the understanding of the biochemical wiring underlying the nitrogen cycle in natural ecosystems. Fluctuating soil redox conditions are indeed a primary feature of many terrestrial ecosystems. Especially in humid tropical forest soil, high net primary productivity and daily rainfall (3.5–4.5 m annually) lead to high availability of reductant and variable redox conditions that cause rapid fluctuation between oxic and anoxic states on a scale of hours to days [7,8]. Similarly, in wetland ecosystems the consumption of oxygen during the night leads to a diurnal fluctuation of the redox conditions in the rhizosphere, resulting in the coexistence of aerobic and anaerobic microbial communities [9].

**Table 1.** Nitrogen biogeochemical processes, associated reaction pathways and biological structures involved in the enzymatic reactions.

nitrogen biogeochemical processes	genes encoding enzymes with the KEGG IDs and EC numbers	nitrogen transformation pathways	reaction rate symbols	biological structure associated with the biochemical pathways
dissimilatory nitrate reduction to ammonium (DNRA)	K00370 *narG:* nitrate reductase alpha subunit (EC: 1.7.99.4)	NO3−→NO2−∫	*r_1_*	$s_{1}:\;narGHIJ,$ *napAB*
	K00371 *narH:* nitrate reductase beta subunit (EC: 1.7.99.4)			
	K00374 *narI:* nitrate reductase gamma subunit (EC: 1.7.99.4)			
	K00373 *narJ:* nitrate reductase delta subunit (EC: 1.7.99.4)			
	K02567 *napA:* periplasmic nitrate reductase (EC: 1.7.99.4)			
	K02568 *napB:* cytochrome c-type protein (EC: 1.7.99.4)			
	K00362 *nirB:* nitrite reductase (NAD(P)H) large subunit (EC: 1.7.1.4)	${\text{NO}}_{2}^{-}\to {\text{NH}}_{4}^{+}$	*r_2_*	$s_{2}:\;nirBD,\;$ nrfAH
	K00363 *nirD:* nitrite reductase (NAD(P)H) small subunit (EC: 1.7.1.4)			
	K03385 *nrfA:* cytochrome c-552 (EC: 1.7.2.2)			
	K15876 *nrfH:* cytochrome c nitrite reductase small subunit (EC: 1.7.2.2)			
assimilatory nitrate reduction	K00367 *narB:* ferredoxin-nitrate reductase (EC: 1.7.7.2)	${\text{NO}}_{3}^{-}\to {\text{NO}}_{2}^{-}$	*r_3_*	$s_{3}:\;narB,$ *nasAB*
	K00372 *nasA:* assimilatory nitrate reductase catalytic subunit (EC: 1.7.99.4)			
	K00360 *nasB:* assimilatory nitrate reductase electron transfer subunit (EC: 1.7.99.4)			
	K00366 *nirA:* ferredoxin-nitrite reductase (EC: 1.7.7.1)	${\text{NO}}_{2}^{-}\to {\text{NH}}_{4}^{+}$	*r_4_*	$s_{4}:\;nirA$
denitrification	K00370 *narG:* nitrate reductase alpha subunit (EC: 1.7.99.4)	${\text{NO}}_{3}^{-}\to {\text{NO}}_{2}^{-}$	*r_1_*	$s_{1}:\;narGHIJ,\;$ *napAB*
	K00371 *narH:* nitrate reductase beta subunit (EC: 1.7.99.4)			
	K00374 *narI:* nitrate reductase gamma subunit (EC: 1.7.99.4)			
	K00373 *narJ:* nitrate reductase delta subunit (EC: 1.7.99.4)			
	K02567 *napA:* periplasmic nitrate reductase (EC: 1.7.99.4)			
	K02568 *napB:* cytochrome c-type protein (EC: 1.7.99.4)			
	K00368 *nirK:* nitrite reductase (NO-forming) (EC: 1.7.2.1)	${\text{NO}}_{2}^{-}\to \text{NO}$	*r_5_*	$s_{5}:\;nirK,\;$ *nirS*
	K15864 *nirS:* nitrite reductase (NO-forming)/hydroxylamine reductase (EC: 1.7.2.1 1.7.99.1)			
	K04561 *norB:* nitric oxide reductase subunit B (EC: 1.7.2.5)	$\text{NO}\to {\text{N}}_{2}\text{O}$	*r_6_*	$s_{6}:\;norBC$
	K02305 *norC:* nitric oxide reductase subunit C (EC: 1.7.2.5)			
	K00376 *nosZ:* nitric oxide reductase (EC: 1.7.2.4)	${\text{N}}_{2}\text{O}\to {\text{N}}_{2}$	*r_7_*	$s_{7}:\;nosZ$
nitrogen fixation	K02588 *nifH:* nitrogenase iron protein (EC: 1.18.6.1)	N2→NH4+∫	*r_8_*	$s_{8}:\;nifDKH,\;$ *anfG*
	K02586 *nifD:* nitrogenase molybdenum-iron protein alpha chain (EC: 1.18.6.1)			
	K02591 *nifK:* nitrogenase molybdenum-iron protein beta chain (EC: 1.18.6.1)			
	K00531 *anfG:* nitrogenase delta subunit (EC: 1.18.6.1)			
nitrification	K10944 *amoA:* methane/ammonia monooxygenase subunit A (EC: 1.14.18.3 1.14.99.39)	${\text{NH}}_{4}^{+}\to {\text{NH}}_{2}\text{OH}$	*r_9_*	$s_{9}:\;amoABC$
	K10945 *amoB:* methane/ammonia monooxygenase subunit B (EC: 1.14.18.3 1.14.99.39)			
	K10946 *amoC:* methane/ammonia monooxygenase subunit C (EC: 1.14.18.3 1.14.99.39)			
	K10535 *hao:* hydroxylamine dehydrogenase (EC: 1.7.2.6)	${\text{NH}}_{2}\text{OH}\to {\text{NO}}_{2}^{-}$	*r_10_*	$s_{10}:\;hao$
	K00370 *narG:* nitrate reductase alpha subunit (EC: 1.7.99.4)	${\text{NO}}_{2}^{-}\to {\text{NO}}_{3}^{-}$	*r_11_*	$s_{11}:\;nor$
	K00371 *narH:* nitrate reductase beta subunit (EC: 1.7.99.4)			
anammox	…. …… hzo: hydrazine oxidoreductase^a^	${\text{NH}}_{4}^{+}\to {\text{N}}_{2}$	*r_12_*	$s_{12}:\;hzo$

^a^KEGG ID and EC number is not assigned in the KEGG database.

**Figure 1.**
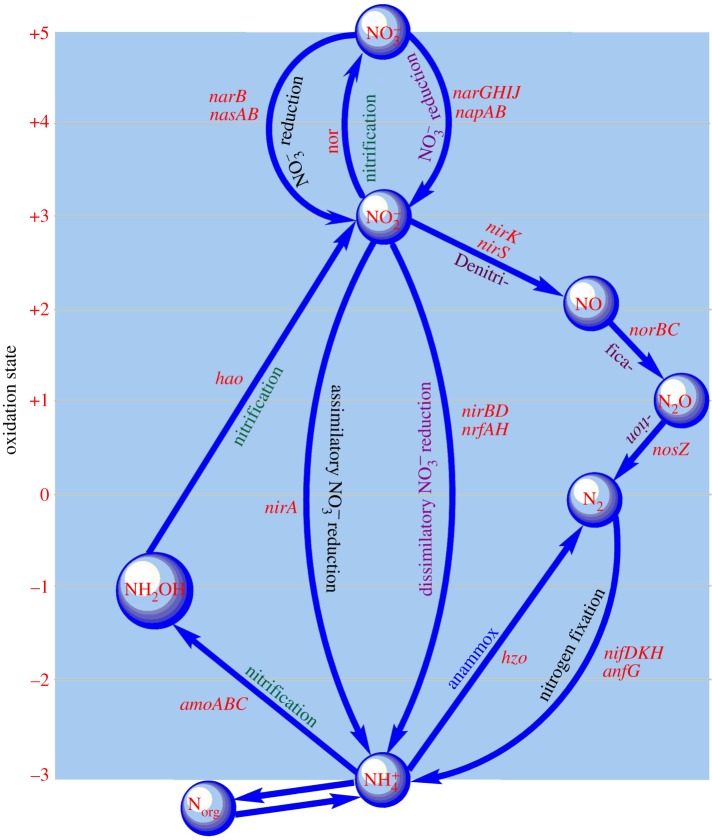
A nitrogen biochemical network constructed with the use of the KEGG nitrogen metabolism database. It consists of interconnected biogeochemical pathways (dissimilatory nitrate reduction to ammonium (DRNA), assimilatory nitrate reduction, denitrification, biological nitrogen fixation (BNF), nitrification and anammox) serving to biochemically process nitrogenous metabolites and transfer them to the next reaction in the network, with the network deficiency zero. There are a variety of genes encoding enzymes that catalyse the important transformation reactions of various nitrogen forms ranging from the oxidation states +5 in nitrate to −3 in ammonium. The ammonium and nitrite act as the network hubs, connecting a relatively maximum number of reactions either as substrate or product of the biochemical reactions.

The complexity of the biochemical network underlying the nitrogen cycle is particularly high [10], as often the end product of one reaction is the substrate of a set of kinetically different, and redox-sensitive, enzymatic reactions. Furthermore, an intriguing feature of the N-cycle is that many of the microbial enzymes involved are pleiotropic: they have multiple regulatory effects, providing either feedback or feed-forward control [11,12]. For example, the enhanced activity of dissimilatory nitrate reductase (e.g. *narG*) gives a positive feedback to a nitrification reaction catalysed by nitrite oxidoreductase (*nor*) and also provides feed-forward inputs to multiple reactions of DNRA and denitrification (figure 1). Understanding these feedback/feed-forward control mechanisms and their evolutionary dynamics is paramount to deepen our understanding of the nitrogen cycle in natural environments. One essential step in this direction is to study *detailed balance* in reaction systems by equilibrating each elementary process by its reverse process. Recent development in complex network theory provides a variety of efficient graph-based techniques to deal with this type of problem [13–15]. Although recent advancements in microbial genomics have provided details on the full set of the enzymes and associated genes [16,17] involved in the nitrogen cycle, many fundamental aspects of the biochemical pathways and their function and interaction in fluctuating redox conditions [18] remain elusive. In particular, it is well understood that fluctuating soil redox conditions facilitate the co-occurrence of different microbial nitrogen transformation pathways with notably different sensitivities to oxygen availabilities [6]. However, how such co-occurrences are maintained and switched in the dominance of one to another pathway in response to the changes in soil redox conditions remains to be understood.

Accelerated human activity during the past century has doubled the annual transfer of nitrogen from the unavailable atmospheric pool to the biologically available forms [19]. The major source of this supply includes heavy use of nitrogen fertilizers, and the combustion of fossil fuels. How it affects terrestrial ecosystems, which are mostly nitrogen limited, is poorly understood. In order to address this broad question, it will be important to know how the nitrogen cycle responds to these changes and maintains its core cycling process [20]. For this purpose, we have investigated the role of alternative biochemical nitrogen transformation pathways in maintaining nitrogen availability in fluctuating redox conditions in the soil. We have introduced a systems approach to model the biogeochemistry of the nitrogen cycle as a biochemical network, which is capable to cycle nitrogen through the interactions of redox reactions. We have kept the biogeochemical dynamics at the level of biochemical reaction network by assuming that the rates of all N-transformations are controlled by the delivery of fresh substrates (by mass transport), rather than by microbial processing capacity (enzyme turnover) [21]. Two distinct subsystems are functionally characterized that switch the ammonium availability with the changes in the soil redox state. Our results indicate that the biochemical network holds inherent adaptive capacity to stabilize ammonium and nitrate availability, and that the bistability in the formation of ammonium is linked to the transient upregulation of the *amo-hao* mediated nitrification pathway. We have characterized a feedback linking two distinct subsystems that regulate the bistability of ammonium formation in oxic and anoxic conditions. An on/off type switching mechanism is shown to be responsible for the activation/inactivation of biochemical pathways to cope with rapid redox variation. Furthermore, our work confirms that the design and organization of microbial nitrogen pathways are ensuring bioavailability of basic nitrogen forms [22], reinforcing the classic idea that organic and inorganic entities have interacted to form a self-regulating complex system that contributes to maintaining the conditions for life [23,24].

## 2. Methods

### 2.1. The nitrogen biochemical network

A general biochemical regulation network of the nitrogen cycle is constructed based on KEGG (www.genome.jp/kegg) (figure 1 and table 1). The core of this network involves four types of reduction (nitrogen fixation, assimilatory nitrate reduction, DNRA and denitrification) and two oxidation N-transformation pathways (nitrification and anaerobic ammonia oxidation (anammox)), where each pathway consists of several kinetically different reactions catalysed by distinct sets of microbial enzymes (table 1). For the construction of the N-biochemical network, we have not considered the gross mineralization and immobilization rates in the soil, as it has been shown that these two processes do not involve either oxidation or reduction reactions and are therefore unaffected by redox variation [6,7,25–27].

We have established a theoretical representation of the network (figure 1) as a connected graph, with directed path between any pair of nodes. The network nodes (closed circles) represent nitrogenous substrates and the directed edges between the nodes symbolize enzymatic reactions that process and transform the nitrogenous substrates. In the network, ammonium and nitrite act as network hubs, connecting a large number of nodes in the network.

**Figure 2.**
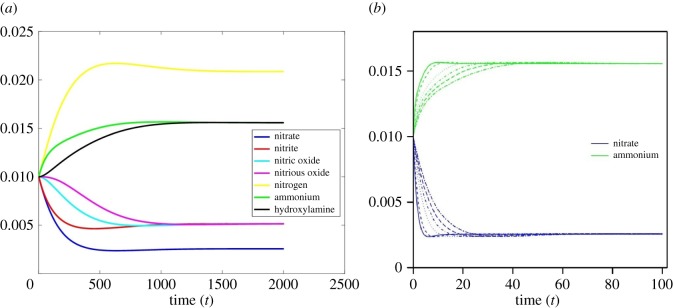
(*a*) Evolution of nitrogen biochemical system: it shows that the system evolves towards a steady state at which each N-metabolite maintains its fixed concentration, illustrating the theoretical prediction of the dynamics of N-network with the deficiency zero; (*b*) steady-state dynamics of ammonium and nitrate: it shows that increased Michaelis constant, *k*_m_, prolongs the transient dynamics before reaching the steady state.

The network consists of 12 reactions denoted by the rate symbols, *r*_1_, *r*_2_, … , *r*_12_. For each reaction, educts and products are considered as nodes associated with the biological structure denoted by the symbols *s*_1_, *s*_2_, … , *s*_12_. This network is *weakly reversible* in the sense that if there exists a path from *C_i_* to *C_j_* then it implies that there exists a path from *C_j_* to *C_i_*. The deficiency property of the network shows that the network holds zero deficiency. The deficiency of the network (*δ*) is calculated by *δ* = *m *− *l *− *s*, where *m* is the number of stoichiometrically distinct complexes, *l* is the number of linkage classes and *s* is the dimension of the stoichiometric subspace. For the nitrogen biochemical network, the number of nitrogen compounds (i.e. number of nodes) determines *m* = 7, the number of connected graphs in the network is *l* = 1, the rank of the stoichiometric matrix, *S*, is 6. The deficiency zero theorem [28] asserts that the reaction system has a unique equilibrium point, which is asymptotically stable. Numerical simulations of the associated mass balance equations, which are described in the following sections, illustrate that the nitrogen biochemical system can eventually reach a steady state at which each reactant maintains a fixed concentration determined by the reaction constants and the concentration of other metabolites (figure 2*a*).

### 2.2. The nitrogen biochemical system

The nitrogen biochemical system comprises all the mass balance equations that are formulated based on the nitrogen biochemical network. The evolution of the system is described by the time-dependent dynamics of all nitrogenous substrate concentrations and it illustrates the steady-state relationship among the associated pathways. The biochemical system is given by,

2.1 $$\frac{\text{d}X}{\text{d}t}=S.v(X),$$

where d*X*/d*t* denotes the time derivative of metabolite concentration, *X*, in the network, which describes instantaneous changes in simultaneously occurring biochemical reactions. *S* is the stoichiometric matrix representing the network topological structure consisting of +1, −1 and 0, where +1 denotes a direct feed-forward connection, −1 a feedback connection and 0 no connections between the two adjacent nitrogenous metabolites. The substrate-dependent enzymatic reaction rate v(X) is described by the irreversible Michaelis–Menten kinetics. The stoichiometric matrix *S* is given by

$$\begin{array}{cc} & \begin{array}{cccccccccccc} r_{1}\; & r_{2}\; & r_{3}\; & r_{4}\; & r_{5}\; & r_{6}\; & r_{7}\; & r_{8}\; & r_{9}\; & r_{10}\; & r_{11}\; & r_{12}\; \end{array}\\ S & =\left(\begin{array}{cccccccccccc} -1 & 0 & -1 & 0 & 0 & 0 & 0 & 0 & 0 & 0 & 1 & 0\\ 1 & -1 & 1 & -1 & -1 & 0 & 0 & 0 & 0 & 1 & -1 & 0\\ 0 & 0 & 0 & 0 & 1 & -1 & 0 & 0 & 0 & 0 & 0 & 0\\ 0 & 0 & 0 & 0 & 0 & 1 & -1 & 0 & 0 & 0 & 0 & 0\\ 0 & 0 & 0 & 0 & 0 & 0 & 1 & -1 & 0 & 0 & 0 & 1\\ 0 & 1 & 0 & 1 & 0 & 0 & 0 & 1 & -1 & 0 & 0 & -1\\ 0 & 0 & 0 & 0 & 0 & 0 & 0 & 0 & 1 & -1 & 0 & 0 \end{array}\right)\begin{array}{c} x_{1}\\ x_{2}\\ x_{3}\\ x_{4}\\ x_{5}\\ x_{6}\\ x_{7} \end{array} \end{array}$$

A system of ordinary differential equations describing time-dependent simultaneous changes of metabolite concentration $x_{1}(t),x_{2}(t),x_{3}(t),x_{4}(t),x_{5}(t),x_{6}(t)$ and *x*_7_(*t*) can be constructed from the vector S.v as follows:

$$\frac{\text{d}X}{\text{d}t}=\left(\begin{array}{c} {\dot{x\;}\;}_{1}\\ {\dot{x\;}\;}_{2}\\ {\dot{x\;}\;}_{3}\\ {\dot{x\;}\;}_{4}\\ {\dot{x\;}\;}_{5}\\ {\dot{x\;}\;}_{6}\\ {\dot{x\;}\;}_{7} \end{array}\right)=S.v=\left(\begin{array}{cccccccccccc} -1 & 0 & -1 & 0 & 0 & 0 & 0 & 0 & 0 & 0 & 1 & 0\\ 1 & -1 & 1 & -1 & -1 & 0 & 0 & 0 & 0 & 1 & -1 & 0\\ 0 & 0 & 0 & 0 & 1 & -1 & 0 & 0 & 0 & 0 & 0 & 0\\ 0 & 0 & 0 & 0 & 0 & 1 & -1 & 0 & 0 & 0 & 0 & 0\\ 0 & 0 & 0 & 0 & 0 & 0 & 1 & -1 & 0 & 0 & 0 & 1\\ 0 & 1 & 0 & 1 & 0 & 0 & 0 & 1 & -1 & 0 & 0 & -1\\ 0 & 0 & 0 & 0 & 0 & 0 & 0 & 0 & 1 & -1 & 0 & 0 \end{array}\right).\left(\begin{array}{c} v_{1}\\ v_{2}\\ v_{3}\\ v_{4}\\ v_{5}\\ v_{6}\\ v_{7}\\ v_{8}\\ v_{9}\\ v_{10}\\ v_{11}\\ v_{12} \end{array}\right),$$

where *x*_1_(*t*): nitrate ${\text{NO}}_{3}^{-},$ *x*_2_(*t*): nitrite ${\text{NO}}_{2}^{-},$ *x*_3_(*t*): nitric oxide NO, *x*_4_(*t*): nitrous oxide N_2_O, *x*_5_(*t*): nitrogen N_2_, *x*_6_(*t*): ammonium ${\text{NH}}_{4}^{+}\text{, }$ *x*_7_(*t*): hydroxylamine NH_2_OH denote the molar concentrations of metabolites. The reaction constants $k_{1},k_{2},k_{3},k_{4},k_{5},k_{6},k_{7},k_{8},k_{9},k_{10},k_{11}$ and *k*_12_ are the reaction rate coefficients for the biochemical reactions $r_{1},r_{2},r_{3},r_{4},r_{5},r_{6},r_{7},r_{8},r_{9},r_{10},r_{11}$ and *r*_12_, respectively (table 2). As we are interested in simulating the qualitative dynamics of the network, we assume, for simplicity, that the associated reaction rates, $v_{i}(X),$ follow irreversible Michaelis–Menten form, $k_{i}X/(k_{{\text{m}}_{i}}+X),(i=1,2,\ldots ,12)$ with the half-saturation coefficient $k_{{\text{m}}_{i}}$. Therefore, the transient behaviour of the metabolite concentrations is described by the following system of ordinary differential equations:

$$\begin{array}{rl} \frac{\text{d}x_{1}}{\text{d}t} & =-v_{1}-v_{3}+v_{11}=-k_{1}\frac{x_{1}}{k_{{\text{m}}_{1}}+x_{1}}-k_{3}\frac{x_{1}}{k_{{\text{m}}_{3}}+x_{1}}+k_{11}\frac{x_{2}}{k_{{\text{m}}_{11}}+x_{2}}\\ \frac{\text{d}x_{2}}{\text{d}t} & =v_{1}-v_{2}+v_{3}-v_{4}-v_{5}+v_{10}-v_{11}\\ & =k_{1}\frac{x_{1}}{k_{{\text{m}}_{1}}+x_{1}}-k_{2}\frac{x_{2}}{k_{{\text{m}}_{2}}+x_{2}}+k_{3}\frac{x_{1}}{k_{{\text{m}}_{3}}+x_{1}}-k_{4}\frac{x_{2}}{k_{{\text{m}}_{4}}+x_{2}}-k_{5}\frac{x_{2}}{k_{{\text{m}}_{5}}+x_{2}}\\ & \;+k_{10}\frac{x_{7}}{k_{{\text{m}}_{10}}+x_{7}}-k_{11}\frac{x_{2}}{k_{{\text{m}}_{11}}+x_{2}}\\ \frac{\text{d}x_{3}}{\text{d}t} & =v_{5}-v_{6}=k_{5}\frac{x_{2}}{k_{{\text{m}}_{5}}+x_{2}}-k_{6}\frac{x_{3}}{k_{{\text{m}}_{6}}+x_{3}}\\ \frac{\text{d}x_{4}}{\text{d}t} & =v_{6}-v_{7}=k_{6}\frac{x_{3}}{k_{{\text{m}}_{6}}+x_{3}}-k_{7}\frac{x_{4}}{k_{{\text{m}}_{7}}+x_{4}}\\ \frac{\text{d}x_{5}}{\text{d}t} & =v_{7}-v_{8}+v_{12}=k_{7}\frac{x_{4}}{k_{{\text{m}}_{7}}+x_{4}}-k_{8}\frac{x_{5}}{k_{{\text{m}}_{8}}+x_{5}}+k_{12}\frac{x_{6}}{k_{{\text{m}}_{12}}+x_{6}}\\ \frac{\text{d}x_{6}}{\text{d}t} & =v_{2}+v_{4}+v_{8}-v_{9}-v_{12}=k_{2}\frac{x_{2}}{k_{{\text{m}}_{2}}+x_{2}}+k_{4}\frac{x_{2}}{k_{{\text{m}}_{4}}+x_{2}}+k_{8}\frac{x_{5}}{k_{{\text{m}}_{8}}+x_{5}}-k_{9}\frac{x_{6}}{k_{{\text{m}}_{9}}+x_{6}}-k_{12}\frac{x_{6}}{k_{{\text{m}}_{12}}+x_{6}}\\ \frac{\text{d}x_{7}}{\text{d}t} & =v_{9}-v_{10}=k_{9}\frac{x_{6}}{k_{{\text{m}}_{9}}+x_{6}}-k_{10}\frac{x_{7}}{k_{{\text{m}}_{10}}+x_{7}} \end{array}$$

**Table 2.** Definition and measuring units of model variables and parameters.

model variables			
variable name	variable definition	units	
*x_1_*	nitrate ${\text{NO}}_{3}^{-}$	mol.l^−1^	
*x_2_*	nitrite ${\text{NO}}_{2}^{-}$		
*x_3_*	nitric oxide NO		
*x_4_*	nitrous oxide N_2_O		
*x_5_*	nitrogen N_2_		
*x_6_*	ammonium ${\text{NH}}_{4}^{+}$		
*x_7_*	hydroxylamine NH_2_OH		

model parameters			
parameter name	reaction pathways	definition	units
*k_1_*	${\text{NO}}_{3}^{-}\to {\text{NO}}_{2}^{-}$	reaction rate coefficients	h^−1^
*k_2_*	${\text{NO}}_{2}^{-}\to {\text{NH}}_{4}^{+}$		
*k_3_*	${\text{NO}}_{3}^{-}\to {\text{NO}}_{2}^{-}$		
*k_4_*	${\text{NO}}_{2}^{-}\to {\text{NH}}_{4}^{+}$		
*k_5_*	${\text{NO}}_{2}^{-}\to \text{NO}$		
*k_6_*	$\text{NO}\to {\text{N}}_{2}\text{O}$		
*k_7_*	${\text{N}}_{2}\text{O}\to {\text{N}}_{2}$		
*k_8_*	${\text{N}}_{2}\to {\text{NH}}_{4}^{+}$		
*k_9_*	${\text{NH}}_{4}^{+}\to {\text{NH}}_{2}\text{OH}$		
*k_10_*	${\text{NH}}_{2}\text{OH}\to {\text{NO}}_{2}^{-}$		
*k_11_*	${\text{NO}}_{2}^{-}\to {\text{NO}}_{3}^{-}$		
*k_12_*	${\text{NH}}_{4}^{+}\to {\text{N}}_{2}$		
*k*_m_	half-saturation coefficient	Michaelis–Menten	micro molar (μM)

Standard Runge–Kutta method of the fourth order is implemented for the computation which runs for the 2001 time steps with delta T, 0.01. Series of simulations with randomized parameters and initial conditions have been run to document dynamic changes of the bioavailable nitrogen forms, ammonium and nitrate: (i) in the first step, we have investigated qualitative changes from ammonium to nitrate rich system and vice versa, with the variation in rate-limiting reaction coefficients, and (ii) in the second step, we have simulated dynamic responses to the external supply of ammonium as step inputs. We have then determined the qualitative and quantitative nature of the point of transition from natural responses, with multiple levels of external ammonium inputs (near to zero, 5-, 10- and 15-fold increased levels). The numerical simulation has been carried out in Matlab v. 7.12.0 (R2011a)

## 3. Results

### 3.1. General dynamic characteristics of the N-biochemical system

The N-biochemical network is a connected graph with zero deficiency, in which ammonium and nitrite act as network hubs connecting relatively a large number of nodes. Dynamic characteristics of this network are critically dependent on the network topology as well as the kinetically associated biochemical systems. Within this network, alteration of biochemical pathways and maintenance of the conservation cycles essentially depend on the soil redox state. The deficiency zero theorem and the numerical simulation of N-biochemical system with randomly chosen parameter values from uniform distribution assert that the nitrogen biochemical system evolves towards a steady state at which the rates of change of nitrogen metabolites, d*X*/d*t*, are zero but at the same time the net rates are non-zero (figure 2*a*). At the steady state, metabolite concentrations are unchanging; however, there are net flows of energy and mass within the system and with the environment. Systems at steady state must therefore be open and will necessarily continuously dissipate any gradients between the system and the external environment. This means that one or more S.v must be non-zero. This is clearly being seen in the analytical calculation of dependent and independent reactions (electronic supplementary material, appendix A). It has been noted that this qualitative property of the steady state remains invariant with respect to the changes of system parameters and initial conditions. It is, however, noted that the variation in Michaelis constant affects the transient period. In particular, increased Michaelis constant, *k*_m_, prolongs the transient dynamics before reaching to the steady state (figure 2*b*; electronic supplementary material, table S2).

In order to determine the mass conversion and conservation characteristics, dependence and independence of the biochemical reactions have been evaluated with the use of the matrix equation, S.v=I.(dX/dt), where *I* is the identity matrix. We have then applied elementary row operations both sides of the matrix equation until the stoichiometric matrix, *S*, is reduced to the echelon form, $\left[\begin{array}{cc} I & A\\ 0 & 0 \end{array}\right]$ (electronic supplementary material, appendix A). The matrix equation is, therefore, converted into the form, $\left[\begin{array}{cc} I & A\\ 0 & 0 \end{array}\right]v=\left[\begin{array}{c} U\\ V \end{array}\right]\frac{\text{d}X}{\text{d}t}$. The conservation cycle is then derived from the equation V(dX/dt)=0 (electronic supplementary material, appendix A). The dependence equation established a connection among the rates of change that do not change in time (i.e. $x_{1}^{\prime }+x_{2}^{\prime }+x_{3}^{\prime }+x_{4}^{\prime }+x_{5}^{\prime }+x_{6}^{\prime }+x_{7}^{\prime }=0$). This is the only conserved cycle that appeared as a linearly dependent row in the stoichiometric matrix; this conservation relationship indicates that the redox pathways are organized in such a way that the N-biochemical network has remained capable of maintaining a flow of nitrogenous substrates to all the enzymatic reactions, retaining a complete N-cycle.

### 3.2. Flipping of bioavailable nitrogen forms

**Figure 3.**
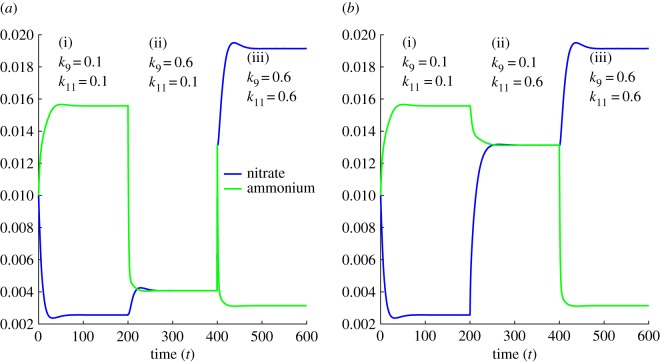
Flipping of ammonium and nitrate availability. (*a*) Dynamic regime (i) (ammonium rich regime) *k_i_* = 0.1, *i* = 1, … ,12; dynamic regime (ii) (nitrogen poor regime) *k_i_* = 0.1, *k*_9_ = 0.6; dynamic regime (iii) (nitrate rich regime) *k_i_* = 0.1, *k*_9_ = 0.6, *k*_11_ = 0.6. (*b*) Dynamic regime (i) and (iii) are the same as (*a*), whereas the dynamic regime (ii) (*b*) is different with the nearly similar concentrations of ammonium and nitrate, *k_i_* = 0, *k*_11_ = 0.6. The nitrogen biochemical system is simulated under the initial conditions *x_j_*(0) = 0.01, *j* = 1, … , 7 and *k*_m_ = 1.

In the absence of external stimuli (e.g. nitrogen fertilization), transient dynamics of ammonium and nitrate concentration follows very different trajectories (figure 3). We have considered the role of soil redox variation implicitly for describing network dynamics. In anoxic redox state, anaerobic microbial pathways are active, whereas aerobic microbial pathways are active in the oxic redox state. We have then simulated the dynamic interchange between these two fundamentally different sets of pathways and subsequently determined the changes in the ammonium and nitrate concentration. While the ammonium concentration increases (decreases), the nitrate concentration decreases (increases) and then both the concentrations finally attain their steady state at which ammonium concentration remains higher (lower) than the nitrate concentration (figure 3*a*(i,iii),*b*(i,iii)). On the other hand, while both ammonium and nitrate concentrations initially decrease (increase) by maintaining slightly lower concentration of ammonium, the nitrate concentration jumps to an elevated level after a certain period of time and then the concentration differences shrink gradually over a longer period of time that eventually establish almost the same ammonium and nitrate concentrations at the steady state (figure 3*a*(ii),*b*(ii)). It, therefore, indicates that nitrogen biochemical network involves a flipping mechanism that can flip the steady-state concentration levels of ammonium and nitrate.

Numerical simulations of the dynamic regulation of the total ammonium and nitrate concentrations have shown that the two rate-limiting steps of the nitrification process, ammonium → hydroxylamine and nitrite → nitrate, play critical roles. While the steady-state level of ammonium is higher than the nitrate, at least sixfold increased rate of conversion from ammonium to hydroxylamine lowers the ammonium concentration at the level of nitrate, resulting in nearly similar concentrations of both the nitrogen forms. Analogously, sixfold increased rate of the conversion from nitrite to nitrate elevates the nitrate at the level of ammonium while keeping all other things unchanged (electronic supporting material). Flipping from an ammonium to nitrate rich system is resulted in at least sixfold increased conversion rates of both the nitrification pathways, ammonium → hydroxylamine and nitrite → nitrate. This happens to be the case as ammonium level decreases continuously through the increased conversion of ammonium → hydroxylamine, with simultaneous increase in nitrate concentration through the elevated conversion rate of nitrite → nitrate.

### 3.3. Bistability of ammonium formation

**Figure 4.**
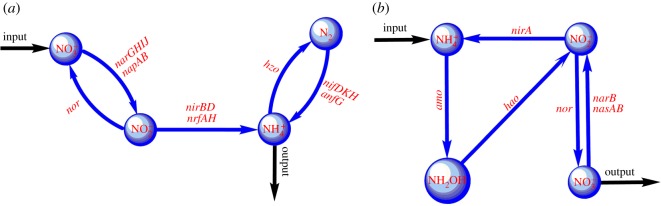
(*a*) Graph of the ammonium-source system: this system reduces dinitrogen and nitrate to ammonium through biochemical processes of BNF and DNRA, respectively. These ammonium generating processes are counterbalanced by the anammox and the second rate-limiting step of nitrification, resulting in anoxic ammonium stable state. (*b*) Graph of the ammonium-sink system: this system consumes ammonium through assimilatory nitrate reduction pathways for the biosynthesis of amino acids and nucleotides. These ammonium-sink pathways are sharply counterbalanced by the nitrification pathways that eventually convert ammonium into nitrate. Thus, the sink systems generally function as stable ammonium sink primarily in oxic soil conditions.

To better understand how ammonium-N formation is regulated through the biochemical pathways, we have studied the dynamic interactions between anaerobic and aerobic biochemical pathways. Dominance of anaerobic or aerobic pathways essentially depends on the anoxic or oxic soil redox state, respectively. While the one set of anaerobic biochemical pathways contribute to producing and stabilizing inorganic ammonium availability, the other set of alternative pathways acts as ammonium sink through nitrogen assimilation and nitrification processes. By separating and removing associated oxic and anoxic biochemical reactions in the same N-transformation pathways, we have designed ammonium-source and -sink subsystems embedded within the whole N-biochemical systems (figure 4*a,b*). These subsystems are basically network modules with monotonicity properties (i.e. every undirected loop in the subgraph has an even number or zero of negative sign) [29]. The monotonicity property ensures the dynamic ‘source’ or ‘sink’ characteristics of the module (electronic supporting material). To understand the subsystem dynamics and interlink between them, we have adopted the classic monotone dynamical systems approach. The idea of monotone dynamical systems, pioneered by Hirsch & Smith [30,31], has shown promising outcomes for decoding the control mechanism of a complex biochemical network, drawing conclusions about control behaviour based only on network topological structures (electronic supporting material). Dynamic monotonicity reflects that a system responds consistently to perturbations on its components and the system trajectories slide along a continuum of steady states. This monotonicity phenomenon is often found in the core of biochemical regulatory networks [32]. It has been observed that the N-biochemical network, which actually forms a non-monotone system, consists of two monotone subsystems that act as either source or sink of ammonium availability.

The ammonium-source system (figure 4*a*) is responsible for releasing and stabilizing inorganic ammonium availability in the soil. By contrast, the ammonium-sink system (figure 4*b*) contributes to the loss of ammonium through the pathways of nitrification and nitrogen assimilation process. These two subsystems share ammonium, nitrite and nitrate as common nitrogenous compounds. The biochemical pathways connecting these compounds in the source systems are kinetically different from the corresponding alternative pathways in the sink systems, excluding a nitrification pathway from nitrite to nitrate which has been shared by both the systems. While the source system includes all N-transformation pathways of DNRA, biological nitrogen fixation (BNF), the last rate-limiting step of nitrification and anammox, the sink system involves all the nitrification and assimilatory biochemical reaction pathways. It should be noted that the ammonium-source pathways are mainly anoxic (excluding the common nitrification pathway), whereas the ammonium-sink processes are oxic in nature.

**Figure 5.**
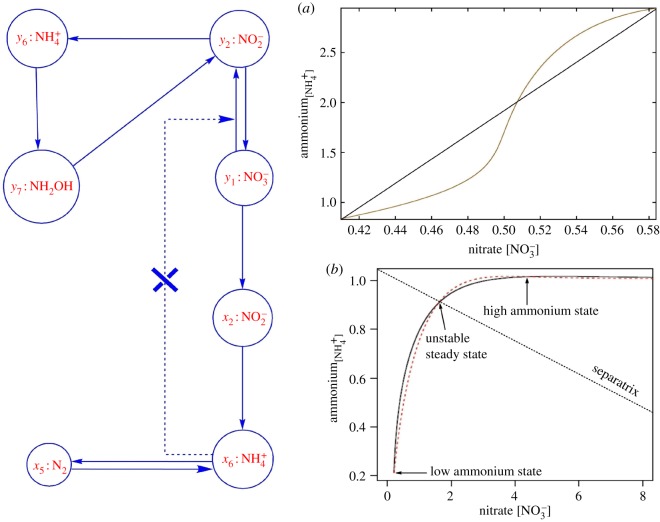
Bistability arises through a positive feedback interconnection between the ammonium-source (*X*-variable) and -sink subsystems (*Y*-variable): the positive interconnection (dotted line) between these two monotone subsystems is defined by feeding the output ammonium of the source system to the sink system as an input; (*a*) the open-loop, feedback-blocked system is monotone and possesses a sigmoidal characteristic which guarantees to have bistability in the system for some range of feedback strengths, (*b*) bistability of the feedback system emerges through the transient augmentation of ammonia oxidizing feedback connectivity. Availability of ammonium is flipped between the low and high concentration stable states and it exhibits a switching threshold. Switching between the lower and higher ammonium state is accompanied by transiently augmenting ammonia oxidation that rapidly reduces ammonium concentration.

**Figure 6.**
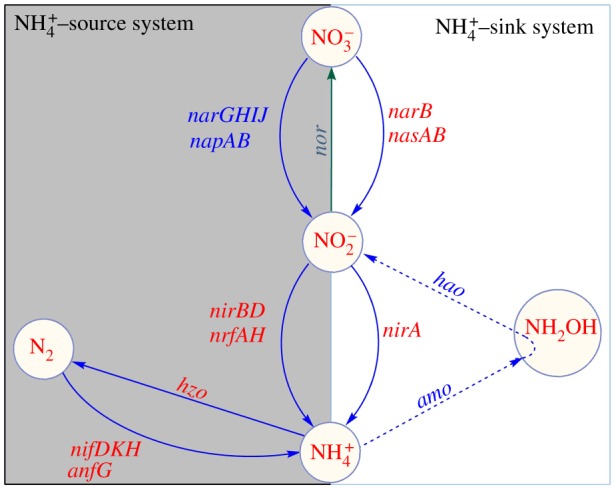
Design and organization of microbial pathways for the bistable control of nitrogen biochemical network: bistable control has emerged through a positive feedback connection between the ammonium-source and -sink systems that underlie the observed biphasic response of the nitrogen biochemical network. This biphasic response has emerged through transient reduction of inorganic ammonium concentration that promotes activities of assimilatory nitrate reductase by relaxing ammonium's inhibitory effects on assimilatory nitrate reductase. Once it crosses the inhibitory threshold, assimilatory pathways contribute more to ammonium stabilization relative to DNRA.

Bistable control [33] of ammonium formation has emerged through the interconnections between ammonium-source and -sink systems (figures 5 and 6). The ‘positive feedback interconnection’ of the system is formally defined by letting the output (i.e. ammonium is an output of the source system and the nitrate is an output of the sink system) of each of them serve as the input of the other [32]. Bistability is largely achieved through the transient upregulation of *amo-hao* and *nar/nap* mediated biochemical pathways (electronic supplementary material). This modulation leads to bistability of the systems. Ammonium availability is flipped between high and low stable states through the transient augmentation of *amo-hao* mediated pathways of nitrification. Without augmentation and/or suppression, monostability is observed. Depending on the soil redox state, this monostability is either established by the sink systems in oxic phase or by the source system in anoxic phase (electronic supplementary material, appendices B and C). Flipping is accomplished by transiently augmenting the *amo-hao* pathway which promotes assimilatory nitrate reductions by reducing the ammonium availability. As dissimilatory nitrate reduction is not affected by ammonium whereas assimilatory nitrate reduction is strongly inhibited by ammonium [34], this augmentation rapidly converts ammonium into nitrite, causing enhanced activity of assimilatory nitrate reductase.

We have further studied the qualitative responses of the source and sink systems to step inputs in the formation of ammonium, with the underlying assumption that both systems will receive ammonium from external sources (e.g. N-fertilization) (electronic supplementary material). Both the systems show quick responses to the inputs. We found that ammonium formation in the source system strictly increases following the stimulus step (bottom-up direction) and eventually converges to the steady state, while the formation strictly tracks the gradually increased inputs in the bottom-up direction. Following the deterrent step input (top-down direction), ammonium formation displays unimodal response with a sharp peak at the deterrent point. When the input decreases gradually with a constant slope, the response curve loses the sharp peak with the curvature determined by the slope of the input functions. By contrast, the sink system displays similar responses to stimulus and deterrent step inputs; while the ammonium formation increases steadily irrespective of the forms of inputs, nitrate concentration increases and reaches the steady state for all the forms of step inputs. These differential response patterns indicate that the source system is more responsive to external ammonium sources, in the sense that ammonium formation is adjusted through the source pathways following the form and degree of the external ammonium supply, whereas the N-sink system shows mostly uniform responses.

### 3.4. Responses to nitrogen fertilization

Nitrogen fertilization is expected to change soil availability of ammonium and nitrate by altering the rate of nitrogen conversion in the N-biochemical system [35]. We have simulated this effect by adding ammonium as pulse input. Similar *in silico* experiments can also be carried out for the nitrate. To correctly locate the departure point from the biochemical steady state, we have designed two different *in silico* experiments: (i) ammonium is supplied at the very beginning of the evolution of the N-biochemical system or (ii) ammonium is supplied at a transient state away from the steady state. For understanding the qualitative characteristics of the departure point, four dynamic regimes are defined following the flipping behaviour mentioned in the previous section: (i) ammonium concentration is more than five times the concentration of nitrate (i.e. ammonia rich regimes), (ii) low availability of both ammonium and nitrate (i.e. nitrogen poor regimes), (iii) nearly identical concentration of ammonium and nitrate and (iv) nitrate concentration is more than five times the ammonium concentration (i.e. nitrate rich regimes). We have then changed the levels of pulse input of ammonium from near-to-zero to 15-fold increased level.

**Figure 7.**
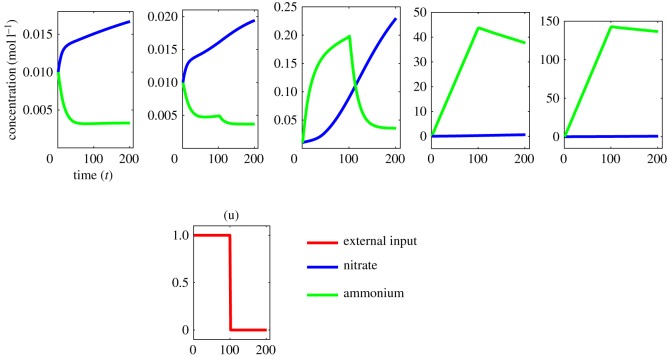
Snapshots of simulated responses of the N-biochemical systems to external ammonium inputs: qualitative changes in ammonium and nitrate levels with the external input level *k* of ammonium (left to right panels), *k* = 0, *k* = 0.001, *k* = 0.1, *k* = 5, *k* = 15 (details are in the electronic supplementary material, text S3, figures S4 and S5 and table S3).

Simulating qualitative and quantitative response of the N-biochemical system to ammonium inputs shows that the system can maintain stable concentrations of ammonium and nitrate with relatively low levels of ammonium input (figure 7). However, fivefold to 15-fold increase in ammonium input completely alters the system's qualitative characteristics. In particular, nitrate rich system and even nitrogen poor system transform into ammonium rich system. With this increased level of inputs, ammonium dynamics departs (either monotonically increases or decreases) from its stable concentration at the point where it starts or stops receiving the inputs (electronic supplementary material, table S3 and figures S4 and S5). It, therefore, indicates that both the quantity and qualitative nature (e.g. periodic or pulse) of nitrogen fertilization do matter for the unstable response behaviour of the N-biochemical system.

## 4. Discussion

Several studies [7,8,9] have clearly indicated how variations in the soil redox potential play a pivotal role in the regulation of the rate and the nature of nitrogen biogeochemical processes. However, the impact of rapid and large redox fluctuations on the N cycling in natural communities remains largely unknown. While classical ecological theory predicts that variable environmental conditions promote high functional diversity and species richness through niche availabilities [36], it fails to explain the coexistence of oxic and anoxic processes in soils where the redox potential shifts faster than the rate of population turnover. In this study, we have shown that the N-biochemical system essentially allows the maintenance of the continuous flow of nitrogenous compounds through the biochemical pathways and the transformation of the molecular N into bioavailable forms. The underlying biochemical mechanisms are an ensemble within a biochemical network holding alternative and reversible biochemical pathways to the primary nitrogen transformation reactions. These alternative biochemical pathways are the key to keep the mass conversion and conservation properties invariant in fluctuating soil redox states.

Soil redox state acts as the principle switch for altering the biochemical pathways, regulating bioavailability of essential nitrogen forms (mainly ammonium and nitrate). In order to understand this regulation, we have simulated the interactions between two counteracting microbial processes, nitrification and nitrate reduction (including DNRA, assimilatory nitrate reduction to ammonium and denitrification) that play a critical role in retaining and restoring the bioavailable nitrogen forms [7,27,37]. The microbial nitrate reduction is a two-step process. In the first step, nitrate is reduced to nitrite and then in the second step nitrite transforms to ammonium. We have studied the effects of dynamic modulation of this reaction rate by both nitrification biochemical pathways (i.e. ammonium → hydroxylamine and nitrite → nitrate) (figure 3). It shows that these two pathways are counteracted to stably maintain ammonium and nitrate concentrations. Increasing conversion rate from ammonium to hydroxylamine lowers the substrate limitation to the intermediate pathways (i.e. hydroxylamine → nitrite) that link ammonium to nitrate. Simultaneous increase in the conversion rate of nitrite → nitrate makes the system nitrate rich relative to ammonium. Similarly, decreasing conversion rate of ammonium → hydroxylamine and subsequent higher limitation to the intermediate pathway contribute to maintaining ammonium rich system. However, it has also been found that proper adjustment of conversion rates of both the pathways can equalize the ammonium and nitrate concentration. At the level of ecosystem, this result indicates that nitrification is an important biogeochemical process playing critical roles for maintaining stable composition of ammonium and nitrate. Similar modulation techniques are practically applied in waste water treatment by tuning environmental conditions that increase the nitrification rate [38].

Three major nitrogen reduction processes in soil compete for the nitrate as electron acceptor: denitrification, DNRA and assimilatory nitrate reduction. Denitrification is an important reduction process reducing nitrate to dinitrogen and nitrous oxide. Only the assimilatory and dissimilatory reduction processes lead to the conversion of nitrate to ammonium. With oxic soil conditions, microbial nitrification converts a large portion of inorganic ammonium into nitrate which consequently runs off into surface waters and causes eutrophication in lake ecosystems. Tuning environmental conditions that can influence the end product of nitrate reduction processes would yield substantial ecological and economic benefits for both natural and engineered ecosystems. It has therefore made the nitrate reduction pathways extraordinarily important in recent ecological and environmental research generating a vast literature based on genomic and metagenomic information [37]. The assimilatory and dissimilatory nitrate reduction processes serve distinct cellular functions: while assimilatory process provides the cell with ammonium required for the synthesis of amino acids and nucleotides, the dissimilatory reduction is a means of generating ATP in absence of oxygen. However, both the processes share the same nitrogen transformation pathways: nitrate to nitrite and then nitrite to ammonium. While some microbial species (e.g. *KlebsielIa aerogenes*) evidently synthesizes a single nitrate reductase protein that can serve an assimilatory or dissimilatory role, others (e.g. *Pseudomonas aeruginosa*) use different enzymes encoded by different genes for assimilatory and dissimilatory reactions [39]. It has never been clearly established how these two processes are regulated and contributed to ammonium availability. In this study, we have illustrated how the end product of these two processes is influenced by the nitrification reaction in fluctuating redox conditions.

It is well established that ammonium is a potent inhibitor of assimilatory nitrate reductase, while the dissimilatory reduction remains unaffected by the ammonium [39]. Further study has unravelled the mechanism by which ammonium inhibits the activity of assimilatory nitrate reductase. It has evidently been illustrated that assimilatory nitrate reduction in soil is strongly regulated by glutamine, a product of intracellular ammonium assimilation, and by the inhibitors of glutamine metabolism (e.g. azaserine, albizziin and aminooxyacetate) [34]. By contrast, dissimilatory nitrate reduction is not affected by ammonium. In tropical forest soils, dissimilatory reduction is observed to be primarily limited by nitrate availability, opposed to carbon or oxygen or ammonium [7]. DNRA organisms appeared to be tolerant to unfavourable oxic conditions, leading to co-occurrence of both assimilatory and dissimilatory reactions in fluctuating soil redox conditions. Here, we have shown biphasic production of ammonium through its inhibitory role. This biphasic characteristic has resulted through the stimulation of the rate of ammonium oxidation, the first step of nitrification. Stimulated ammonium oxidation, leading to quick reduction of available ammonium, has given a positive feedback into assimilatory process. This transient modulation leads to bistability of the system. Ammonium availability is flipped between high and low stable states through the transient augmentation of *amo-hao* mediated pathways of nitrification. Without augmentation, monostability is observed. Flipping is accomplished by transiently augmenting the *amo-hao* pathway which promotes assimilatory nitrate reductions by reducing the ammonium availability. As dissimilatory nitrate reduction is not affected by ammonium whereas assimilatory nitrate reduction is strongly inhibited by ammonium [34], this augmentation rapidly converts ammonium into nitrite, causing enhanced activity of assimilatory nitrate reductase.

In this study, we have clearly demonstrated that the biphasic production of ammonium has been maintained stably through the positive feedback interconnection between the two subsystems (figure 6). Both the subsystems are basically the N-biochemical reaction network modules with distinct functions and holding monotonicity property that guarantees global convergence to steady states, ruling out unpredictable behaviours and even sustained oscillations. The monotone dynamics is maintained throughout no matter what the values of the reaction parameters are and is firmly based only on the wiring structure (electronic supplementary material). While the one subsystem, namely the ‘source system’, ensures stability of ammonium formation mainly in anoxic conditions, the other subsystems named the ‘sink system’ establish ammonium stability in relatively oxic conditions. The source system reduces dinitrogen and nitrate to ammonium through BNF and DNRA processes, respectively. These ammonium generating processes are counterbalanced by the anammox and the second rate-limiting step of nitrification, resulting in anoxic ammonium stable state. By contrast, the sink system consumes ammonium through assimilatory nitrate reduction pathways for the biosynthesis of amino acids and nucleotides. These ammonium-sink pathways are sharply counterbalanced by the nitrification pathways that eventually convert ammonium into nitrate. Thus, the sink systems generally function as ammonium-sink primarily in oxic soil conditions. Positive feedback interconnections between the source and sink systems are established by the internal biochemical pathways of ammonium oxidation in the sink system and the first step of DNRA in the source system that converts nitrate to nitrite. Bistability of the feedback system emerged through the transient augmentation of ammonia oxidizing feedback connectivity. Availability of ammonium is flipped between the low and high concentration stable states and it exhibits a switching threshold. Switching between the lower and higher ammonium state is accompanied by transiently augmenting ammonia oxidation that rapidly reduces ammonium concentration. Transient reduction of inorganic ammonium concentration promotes activities of assimilatory nitrate reductase by relaxing ammonium's inhibitory effects. Once it crosses the inhibitory threshold, assimilatory pathways contribute more to ammonium stabilization relative to DNRA (figure 6). This bistable control mechanism of ammonium formation through ammonium oxidation pathways can explain common situations in tropical forest soil in which ammonium concentration is relatively maintained at a higher level without wider BNF activities [40].

Nitrogen fertilizer is one of the primary anthropogenic sources of non-BNF in managed and engineered ecosystems. A large amount of ammonium fertilizer is converted to nitrate that mostly runs off into surface water and causes eutrophication in costal zones. Beside this nitrification effect, denitrification plays a significant role in releasing nitrous oxide, a potent greenhouse gas, from ammonium fertilizer [41]. In this study, we have asked the question how the N-biochemical system responds to N-fertilization and then how it affects ammonium and nitrate availability in the system. We have simulated the qualitative response through *in silico* experiments. Upon exposure to anthropogenic processes, like nitrogen fertilization and atmospheric nitrogen deposition, the nitrogen biochemical system shifts from stable to an unstable state with steadily increasing rate of ammonium and nitrate in the soil. Consequently, our results suggest that both the magnitude and the qualitative nature of the effect are important for the departure from the natural biochemical steady state. With increasing supply of ammonium nitrogen, ammonium accumulation will be elevated at a much faster rate relative to its conversion rate to hydroxylamine or rate of ammonium loss through anammox reaction. Given this condition, the nitrogen poor system can quickly become ammonium rich system as illustrated by our simulation results. Thus, our results suggest that elevated anthropogenic pressure can breakdown the stable functioning of the nitrogen biochemical system and consequently alter soil availability of ammonium and nitrate, which have dramatic negative consequences for maintaining the condition of life.

Systems biology approaches have evidently shown its tremendous potential in generating novel insights about cellular functions by considering genes and their products as the cells' crucial interacting parts. Parallel to this established approach at the level of cell and molecule, this study has introduced, for the first time, a systems biology-like approach for understanding nitrogen biogeochemistry. The study has shown that the N-cycle involves a complex biochemical system within which the general N-biochemical reactions are wired to transforming and cycling the nitrogenous compounds. Complexity (i.e. alternative wiring structure) has provided explanation to maintaining mass conversion and conservation properties invariant in rapidly changing soil redox conditions. It is predicted that the nitrogen biochemical network involves two counteracting modules with specific input–output and source- and sink-type functional characteristics that allow stabilizing and maintaining bioavailability of ammonium in fluctuating redox states. The results, therefore, have significance in thriving research areas focusing on linking molecule to ecosystems [42], while uncovering natural feedback/feed-forward control mechanisms for (re)designing of biochemical pathways of nitrogen cycle.

## Supplementary Material

Supporting Info: Nitrogen Biogeochemical Network

## Data accessibility

Additional information can be found in the electronic supplementary material.
