# Supplementary material for: Organization of biogeochemical nitrogen pathways with switch-like adjustment in fluctuating soil redox conditions
Source: R Soc Open Sci. 2017 Jan 11;4(1):160768. doi: 10.1098/rsos.160768 (PMC5319346; doi:10.1098/rsos.160768)
Supplement: Supporting Info: Nitrogen Biogeochemical Network [file rsos160768supp1.pdf]

Supporting Information for

Organization of biogeochemical nitrogen pathways with switch-like adjustment in fluctuating soil redox conditions

Sanjay Lamba[1], Soumen Bera[1], Mubasher Rashid[1], Alexander B. Medvinsky[2], Gui-Quan Sun[3], Claudia Acquisti[4], Bai-Lian Li[5], Amit Chakraborty[1,5*]

[1]School of Mathematics, Statistics and Computational Sciences
Central University of Rajasthan, Bandarsindri, Ajmer, India.

[2]Institute of Theoretical and Experimental Biophysics, Pushchino 142290, Russia.

[3]Department of Mathematics, Shanxi University, Taiyuan, China.

[4]Institute for Evolution and Biodiversity, WWU Muenster, Germany.

[5]Ecological Complexity and Modeling Laboratory, University of California, Riverside, CA 92521-0124, USA.

***E-mail: amitc.envsc@yahoo.com , amitc.maths@curaj.ac.in.**

Contents of this file

Text S1 to S10
Figures S1 to S15
Tables S1 to S5

Introduction

This Auxiliary Material includes five supplementary tables (Table S1-S5), fifteen supplementary figures (Figs. S1-S15) and ten supplementary texts(Text S1-S10). Detailed methods and mathematical techniques are described in the supplementary texts; all the simulated model results in the form of tables and figures are presented in the auxiliary figures and tables. This material presents how the nitrogen biochemical system runs and is regulated over time and how it is responded to external ammonium supply.

S1 Nitrogen Biochemical Network

The **nitrogen biochemical network** is constructed with the use of Kyoto Encyclopaedia of Genes and Genome database (KEGG,`www.genome.jp/kegg`). It consists of twelve commonly occurred biochemical pathways that serve to biochemically process nitrogenous metabolites and transfer the product into next reaction of the network (Figure S1).

The network nodes are representing nitrogenous metabolites within a closed circle and a directed edge between the nodes symbolize enzymatic reaction that biochemically processes the metabolites and extract nitrogenous form different from the substrate. A rectangular box on the edge denotes biological structure excreted by a variety of soil microbes. It can be noticed that there are multiple biochemical pathways (edges) that catalyze by different biological structure between the same substrate and product of microbial actions, forming a complex biochemical nitrogen network. In the network, ammonium and nitrite act as network hubs connecting relatively a large number of nodes in the network. The graph representation of this network asserts that it forms a connected graph, as there exists a directed path between any pair of nodes. However, species graph representation of the associated biochemical reactions ensures that the nitrogen biochemical network cannot form a monotone dynamical system because of multiple biochemical pathways between a pair of nodes.

Table S1 is list the biochemical reaction pathways symbolized by $'r'$ and the associated biological structure catalysing the pathway reaction.

S2 Mass Balance Equations of the Nitrogen (N) Biochemical Network

The mass balance equations are formulated for the nitrogen biochemical reactions network which describe the dynamics of all nitrogenous metabolite concentrations and illustrate the steady-state relationship among the associated pathways. The equation is given by,

$$\frac{dX}{dt} = S.v(X),$$

where $\frac{dX}{dt}$ denotes time derivative of the metabolite concentration, X in the network, describing instantaneous change in simultaneously occurring biochemical reactions. S is the stoichiometric matrix representing the network topological structure, in which metabolites are represented by the nodes and the directed edges symbolize unidirectional biochemical reactions. The substrate-dependent enzymatic reaction rate, $v(X)$, is described by the irreversible Michaelis-Menten kinetics. The stoichiometric matrix S is given by

$$S = \begin{array}{c} \begin{matrix} r_1 & r_2 & r_3 & r_4 & r_5 & r_6 & r_7 & r_8 & r_9 & r_{10} & r_{11} & r_{12} \end{matrix} \\ \begin{pmatrix} -1 & 0 & -1 & 0 & 0 & 0 & 0 & 0 & 0 & 0 & 1 & 0 \\ 1 & -1 & 1 & -1 & -1 & 0 & 0 & 0 & 0 & 1 & -1 & 0 \\ 0 & 0 & 0 & 0 & 1 & -1 & 0 & 0 & 0 & 0 & 0 & 0 \\ 0 & 0 & 0 & 0 & 0 & 1 & -1 & 0 & 0 & 0 & 0 & 0 \\ 0 & 0 & 0 & 0 & 0 & 0 & 1 & -1 & 0 & 0 & 0 & 1 \\ 0 & 1 & 0 & 1 & 0 & 0 & 0 & 1 & -1 & 0 & 0 & -1 \\ 0 & 0 & 0 & 0 & 0 & 0 & 0 & 0 & 1 & -1 & 0 & 0 \end{pmatrix} \end{array} \begin{matrix} x_1 \\ x_2 \\ x_3 \\ x_4 \\ x_5 \\ x_6 \\ x_7 \end{matrix} .$$

A system of ordinary differential equations describing time-dependent simultaneous changes of metabolite concentration $x_1(t), x_2(t), x_3(t), x_4(t), x_5(t), x_6(t)$ and $x_7(t)$ can be constructed from the vector $S.v$ as follows:

$$\frac{dX}{dt} = \begin{pmatrix} \dot{x}_1 \\ \dot{x}_2 \\ \dot{x}_3 \\ \dot{x}_4 \\ \dot{x}_5 \\ \dot{x}_6 \\ \dot{x}_7 \end{pmatrix} = S.v = \begin{pmatrix} -1 & 0 & -1 & 0 & 0 & 0 & 0 & 0 & 0 & 0 & 1 & 0 \\ 1 & -1 & 1 & -1 & -1 & 0 & 0 & 0 & 0 & 1 & -1 & 0 \\ 0 & 0 & 0 & 0 & 1 & -1 & 0 & 0 & 0 & 0 & 0 & 0 \\ 0 & 0 & 0 & 0 & 0 & 1 & -1 & 0 & 0 & 0 & 0 & 0 \\ 0 & 0 & 0 & 0 & 0 & 0 & 1 & -1 & 0 & 0 & 0 & 1 \\ 0 & 1 & 0 & 1 & 0 & 0 & 0 & 1 & -1 & 0 & 0 & -1 \\ 0 & 0 & 0 & 0 & 0 & 0 & 0 & 0 & 1 & -1 & 0 & 0 \end{pmatrix} . \begin{pmatrix} v_1 \\ v_2 \\ v_3 \\ v_4 \\ v_5 \\ v_6 \\ v_7 \\ v_8 \\ v_9 \\ v_{10} \\ v_{11} \\ v_{12} \end{pmatrix},$$

where $x_1(t)$: Nitrate NO_3^-, $x_2(t)$: Nitrite NO_2^-, $x_3(t)$: Nitric Oxide NO, $x_4(t)$: Nitrous Oxide N_2O, $x_5(t)$: Nitrogen N_2, $x_6(t)$: Ammonium NH_4^+, $x_7(t)$: Hydroxylamine NH_2OH denote the molar concentrations of metabolites. And the constants $k_1, k_2, k_3, k_4, k_5, k_6, k_7, k_8, k_9$, k_{10}, k_{11} and k_{12} are the reaction rate coefficients for the biochemical reactions $r_1, r_2, r_3, r_4, r_5, r_6, r_7, r_8, r_9, r_{10}, r_{11}$ and r_{12} respectively (Tables S1 & S2). As we are interested in simulating the qualitative dynamics of the network, we assume, for simplicity, that the associated reaction rates, $v_i(X)$, has irreversible Michaelis-Menten form, $\frac{k_i X}{k_{m_i}+X} (i = 1, 2, \ldots, 12)$, with the half-saturation co-efficient k_{m_i}.

Therefore, the transient behaviour of the metabolite concentrations are described by the following system of ordinary differential equations:

$$\begin{aligned}
\frac{dx_1}{dt} &= -v_1 - v_3 + v_{11} = -k_1 \frac{x_1}{k_{m_1} + x_1} - k_3 \frac{x_1}{k_{m_3} + x_1} + k_{11} \frac{x_2}{k_{m_{11}} + x_2} \\
\frac{dx_2}{dt} &= v_1 - v_2 + v_3 - v_4 - v_5 + v_{10} - v_{11} \\
&= k_1 \frac{x_1}{k_{m_1} + x_1} - k_2 \frac{x_2}{k_{m_2} + x_2} + k_3 \frac{x_1}{k_{m_3} + x_1} - k_4 \frac{x_2}{k_{m_4} + x_2} - k_5 \frac{x_2}{k_{m_5} + x_2} + k_{10} \frac{x_7}{k_{m_{10}} + x_7} - k_{11} \frac{x_2}{k_{m_{11}} + x_2} \\
\frac{dx_3}{dt} &= v_5 - v_6 = k_5 \frac{x_2}{k_{m_5} + x_2} - k_6 \frac{x_3}{k_{m_6} + x_3} \\
\frac{dx_4}{dt} &= v_6 - v_7 = k_6 \frac{x_3}{k_{m_6} + x_3} - k_7 \frac{x_4}{k_{m_7} + x_4} \\
\frac{dx_5}{dt} &= v_7 - v_8 + v_{12} = k_7 \frac{x_4}{k_{m_7} + x_4} - k_8 \frac{x_5}{k_{m_8} + x_5} + k_{12} \frac{x_6}{k_{m_{12}} + x_6} \\
\frac{dx_6}{dt} &= v_2 + v_4 + v_8 - v_9 - v_{12} = k_2 \frac{x_2}{k_{m_2} + x_2} + k_4 \frac{x_2}{k_{m_4} + x_2} + k_8 \frac{x_5}{k_{m_8} + x_5} - k_9 \frac{x_6}{k_{m_9} + x_6} - k_{12} \frac{x_6}{k_{m_{12}} + x_6} \\
\frac{dx_7}{dt} &= v_9 - v_{10} = k_9 \frac{x_6}{k_{m_9} + x_6} - k_{10} \frac{x_7}{k_{m_{10}} + x_7}
\end{aligned}$$

.

Numerical simulation of the mass balance equations illustrates that the system can eventually reach to a steady-state at which each reactant maintains a fixed concentration determined by the reaction constants and the concentration of other metabolites (Figure S2). In particular, we observe that the transient dynamics of ammonium and nitrate concentration follow very different

trajectories. (Figure S3), While the ammonium concentration increases (decreases), the nitrate concentration decreases (increases) and then both concentrations finally attain its steady-state at which ammonium concentration remains higher (lower) than the nitrate concentration. On the other hand, while both the concentrations initially decrease by maintaining higher concentration of ammonium, the nitrate concentration jumps to an elevated level after a certain period of time and then concentration difference shrink gradually over a longer period of time. This qualitative behaviour is, therefore, indicating that nitrogen biochemical network involves a switching mechanism that flips the steady-state levels of inorganic ammonium and nitrate- the most common bioavailable forms of nitrogen.

S3 Response of $N-$Biochemical Systems to $N-$Fertilization

We have simulated this effect by adding ammonium as pulse input. Similar in-silico experiment can be carried out for the nitrate as well. To correctly locate the departure point from the biochemical steady-state, we have designed two different in silico experiments: (a) Ammonium is supplied at very beginning of the evolution of $N-$biochemical system, and (b) Ammonium is supplied at a transient state away from the steady-state. For understanding the qualitative characteristics of the departure point, four dynamic regimes are defined following the flipping behavior mentioned in the previous section:- I: Ammonium concentration is more than the 5 times of nitrate concentration (i.e., ammonia rich regimes), II: Low availability of both ammonium and nitrate (i.e., nitrogen poor regimes), III: Nearly identical concentration of ammonium and nitrate, and IV: Nitrate concentration is more than the 5 times of ammonium concentration (i.e., nitrate rich regimes). We have then changed the levels of pulse input of ammonium from near-to-zero to 15 fold increased level(Table S3, Figures S4 & S5).

S4 Design of Monotone Dynamical Systems

Smith [1], Hirsch and Smith [2] have introduced the idea of monotone dynamical systems with the properties of robust dynamical stability and predictability of responses to perturbations. Basic qualitative feature of this system is that its dynamical behaviour depends only on associated network structure not upon the precise values of the parameters or even the forms of reactions. This system describes the evolution of states which are time-dependent vector

$$X(t) = (x_1(t), x_2(t), x_3(t), \ldots, x_n(t)),$$

where component x_i represents concentration of chemical species such as metabolities. Such reaction system has often been represented as autonomous system of ordinary differential equations with the form:

$$\frac{dX}{dt} = S.v(X),$$

where $v(X)$ is a $m-$dimensional vector of reactions and S is an $n \times m$ matrix called stoichiometric matrix.

A graph based approach has been developed to construct monotone dynamical system associated with a biochemical network. Species graph G represents the topological structure of the biochemical network, with all the nodes correspond to chemical species and the directed edges between the nodes symbolize the biochemical reaction. A dynamical system is then said to be monotone if there exists at least one consistent spin assignment for its associated species graph G. Continuous-time monotone systems have convergent behaviour; they cannot admit any possible

stable oscillations. If there is only one steady-state and solutions are bounded, the every solution converges to this unique steady-state. Instead, if there are multiple steady-states, the Hirsch Generic convergence theorem asserts that generic bounded solutions of strongly montone system must converge to the set of steady-states. In particular, no "chaotic" or any other "strange" dynamics can occur (see the details in Sontag [3]).

Positive feedback interconnections between monotone systems with the output of one enter as input to other establish **bistability.** Besides having intuitive proof of bistability for simple production/degradation system with sigmoidal characteristics, this phenomenon has well demonstrated for Mitogen-activated protein kinase (MAPK) cascades [4].

We have adopted Montone dynamical system for decomposing the biochemical nitrogen network. A spin assignment and consistency based graphical approach has been implemented to derive a monotone subsystem, which says that a dynamical system is monotone if and only if there exists at least one consistent spin assignment for its associated reaction graph. In addition, there will be a consistent spin assignment if and only if every undirected loop in the graph has an even number or zero of negative sign. For constructing a monotone dynamical system (Figure S6), following rules are considered that are associated with a monotone graph:

$$\begin{aligned} \frac{dx}{dt} &= -k_1 \frac{x}{k_{m_1} + u} + k_2 \frac{u}{k_{m_2} + u} \\ \frac{du}{dt} &= -k_2^{'} \frac{u}{k_{m_2} + u} + k_1^{'} \frac{x}{k_{m_1} + x} \end{aligned}$$

where the positive terms in the equations describe the formation and the negative terms describe the degradation rate of metabolites. At a steady-state, degradation rate equates with the formation rate which is given by $\frac{dx}{dt} = \frac{du}{dt} = 0$. It shows that steady states lies on the curve $ux = (k_{m_1})(k_{m_2})$.(Figure S7)

Bistability is often detected in biochemical systems that contain a positive feedback loop or double-negative feedback loop. A bistable system exhibits flipping behaviour between two steady states while passing through an unstable steady-state. It has been observed among various biochemical systems with different orders that the presence of at least one positive feedback loop is necessary for the emergence of bistability (Thomas[6]). However, mere presence of positive feedback loop does not guarantee bistability. Indeed, this is one of the common technical problems for detecting bistability in higher order system where phase plane analysis often fails. Using the classic theory of monotone dynamical systems, Angeli [4] has established an elegant method for the analysis of bistability in positive feedback systems of arbitrary order. It showed that if the open-loop, feedback-blocked system is monotone and possesses a sigmoidal response characteristic, then the feedback system always produces bistability for some range of feedback strength.

We have employed this method for explaining observed bistability in ammonium formation in the $N-$biochemical system. Using the graphical method (Sontag[3]), we have designed two monotone subsystems, termed as ammonium- source and sink systems, embedded in the $N-$ biochemical system. Each subsystem produces monostable ammonium state. The source system receives nitrate as input and produces ammonium as an output, whereas the sink system acts in reverse way. A positive-feedback connection between these two subsystems is given by the ammonium oxidation pathways which is mediated by *amo* and *hao* encoded enzymes converting ammonium to hydroxylamine and then to nitrite . Switching from the lower to higher ammonium state is accompanied by transiently augmenting ammonia oxidation that rapidly reduces ammonium

concentration. Transient reduction of inorganic ammonium concentration promotes activities of assimilatory nitrate reductase by relaxing ammonium's inhibitory effects. Once it crosses the inhibitory threshold, assimilatory pathways contribute more to total ammonium formation relative to DNRA.

S5 Ammonium Source Systems

Associated stoichiometric matrix, S_{source}, describing the NH_4^+−source topological structure (Figure S8) is given by

$$
S_{source} = \begin{array}{c} \begin{matrix} r_1 & r_2 & r_8 & r_{11} & r_{12} \end{matrix} \\ \begin{pmatrix} -1 & 0 & 0 & 1 & 0 \\ 1 & -1 & 0 & -1 & 0 \\ 0 & 1 & 1 & 0 & -1 \end{pmatrix} \end{array} \begin{matrix} x_1 \\ x_2 \\ x_6 \end{matrix} .
$$

Montone dynamical system for NH_4^+−source systems is, therefore, written as:

$$
\begin{aligned}
\frac{dx_1}{dt} &= -k_1\frac{x_1}{k_{m_1}+x_1} + k_{11}\frac{x_2}{k_{m_{11}}+x_2} \\
\frac{dx_2}{dt} &= k_1\frac{x_1}{k_{m_1}+x_1} - k_2\frac{x_2}{k_{m_2}+x_2} - k_{11}\frac{x_2}{k_{m_{11}}+x_2} \\
\frac{dx_6}{dt} &= k_2\frac{x_2}{k_{m_2}+x_2} + k_8\frac{x_5}{k_{m_8}+x_5} - k_{12}\frac{x_6}{k_{m_{12}}+x_6}
\end{aligned}
$$

S6 Ammonium Sink Systems

Associated stoichiometric matrix, S_{sink}, describing the NH_4^+−sink topological structure (Figure S11) is given by

$$
S_{sink} = \begin{array}{c} \begin{matrix} r_3 & r_4 & r_9 & r_{10} & r_{11} \end{matrix} \\ \begin{pmatrix} -1 & 0 & 0 & 0 & 1 \\ 1 & -1 & 0 & 1 & -1 \\ 0 & 1 & -1 & 0 & 0 \\ 0 & 0 & 1 & -1 & 0 \end{pmatrix} \end{array} \begin{matrix} x_1 \\ x_2 \\ x_6 \\ x_7 \end{matrix} .
$$

Monotone dynamical system for the NH_4^+−sink pathways is, therefore, written as:

$$
\begin{aligned}
\frac{dx_1}{dt} &= -k_3\frac{x_1}{k_{m_3}+x_1} + k_{11}\frac{x_2}{k_{m_{11}}+x_2} \\
\frac{dx_2}{dt} &= k_3\frac{x_1}{k_{m_3}+x_1} - k_4\frac{x_2}{k_{m_4}+x_2} + k_{10}\frac{x_7}{k_{m_{10}}+x_7} - k_{11}\frac{x_2}{k_{m_{11}}+x_2} \\
\frac{dx_6}{dt} &= k_4\frac{x_2}{k_{m_2}+x_2} - k_9\frac{x_6}{k_{m_9}+x_6} \\
\frac{dx_7}{dt} &= k_9\frac{x_6}{k_{m_9}+x_6} - k_{10}\frac{x_7}{k_{m_{10}}+x_7}
\end{aligned}
$$

.

S7 Simulating Responses of NH_4^+−Source and NH_4^+−Sink Systems to External Step Inputs

We have simulated the responses of NH_4^+−source and NH_4^+−sink systems to step inputs in the formation of ammonium, with the underlying assumption that both the system will receive ammonium from external sources either through N fertilization or biological N fixation that effect microbial nitrogen pathways. Tables (S4 & S5) and the associated diagrams shows the responses of NH_4^+ source-sink systems (Figures S14 & S15). Differential response patterns of these two systems indicate disparities in the roles of NH_4^+−sink and NH_4^+−source subsystems in the nitrogen biochemical network.

S8 Appendix A: Dependent and Independent Biochemical Reactions

With the use of standard matrix method, we have been determined the dependence and independence of biochemical reactions represented by the equation,

$$S.v = I\frac{dX}{dt},$$

where I is Identity Matrix.

We then apply elementary row operations both sides of the above equation until stoichiometric matrix S is reduced to echelon form R, where

$$R = \begin{bmatrix} I & A \\ 0 & 0 \end{bmatrix}.$$

We have

$$R.v = M\frac{dX}{dt} = \begin{bmatrix} U \\ V \end{bmatrix}\frac{dX}{dt},$$

partition the M matrix in to U and V, along the same row line as the reduced echelon form.

$$\text{where} I = \begin{pmatrix} 1 & 0 & 0 & 0 & 0 & 0 & 0 \\ 0 & 1 & 0 & 0 & 0 & 0 & 0 \\ 0 & 0 & 1 & 0 & 0 & 0 & 0 \\ 0 & 0 & 0 & 1 & 0 & 0 & 0 \\ 0 & 0 & 0 & 0 & 1 & 0 & 0 \\ 0 & 0 & 0 & 0 & 0 & 1 & 0 \\ 0 & 0 & 0 & 0 & 0 & 0 & 1 \end{pmatrix}, \frac{dX}{dt} = \begin{pmatrix} \dot{x}_1 \\ \dot{x}_2 \\ \dot{x}_3 \\ \dot{x}_4 \\ \dot{x}_5 \\ \dot{x}_6 \\ \dot{x}_7 \end{pmatrix},$$

$$S = \begin{pmatrix} -1 & 0 & -1 & 0 & 0 & 0 & 0 & 0 & 0 & 0 & 1 & 0 \\ 1 & -1 & 1 & -1 & -1 & 0 & 0 & 0 & 0 & 1 & -1 & 0 \\ 0 & 0 & 0 & 0 & 1 & -1 & 0 & 0 & 0 & 0 & 0 & 0 \\ 0 & 0 & 0 & 0 & 0 & 1 & -1 & 0 & 0 & 0 & 0 & 0 \\ 0 & 0 & 0 & 0 & 0 & 0 & 1 & -1 & 0 & 0 & 0 & 1 \\ 0 & 1 & 0 & 1 & 0 & 0 & 0 & 1 & -1 & 0 & 0 & -1 \\ 0 & 0 & 0 & 0 & 0 & 0 & 0 & 0 & 1 & -1 & 0 & 0 \end{pmatrix}, \text{and} v = \begin{pmatrix} v_1 \\ v_2 \\ v_3 \\ v_4 \\ v_5 \\ v_6 \\ v_7 \\ v_8 \\ v_9 \\ v_{10} \\ v_{11} \\ v_{12} \end{pmatrix}$$

After Echelon Form

$$R.v = I\frac{dX}{dt}$$

where $R = \begin{bmatrix} I & A \\ 0 & 0 \end{bmatrix}$ = Echelon Form of S is

$$\begin{bmatrix} 1 & 0 & 1 & 0 & 0 & 0 & 0 & 0 & 0 & 0 & -1 & 0 \\ 0 & 1 & 0 & 1 & 0 & 0 & 0 & 1 & 0 & -1 & 0 & -1 \\ 0 & 0 & 0 & 0 & 1 & 0 & 0 & -1 & 0 & 0 & 0 & 1 \\ 0 & 0 & 0 & 0 & 0 & 1 & 0 & -1 & 0 & 0 & 0 & 1 \\ 0 & 0 & 0 & 0 & 0 & 0 & 1 & -1 & 0 & 0 & 0 & 1 \\ 0 & 0 & 0 & 0 & 0 & 0 & 0 & 0 & 1 & -1 & 0 & 0 \\ 0 & 0 & 0 & 0 & 0 & 0 & 0 & 0 & 0 & 0 & 0 & 0 \end{bmatrix}, v = \begin{pmatrix} v_1 \\ v_2 \\ v_3 \\ v_4 \\ v_5 \\ v_6 \\ v_7 \\ v_8 \\ v_9 \\ v_{10} \\ v_{11} \\ v_{12} \end{pmatrix}, \frac{dX}{dt} = \begin{pmatrix} \dot{x}_1 \\ \dot{x}_2 \\ \dot{x}_3 \\ \dot{x}_4 \\ \dot{x}_5 \\ \dot{x}_6 \\ \dot{x}_7 \end{pmatrix}$$

and $M = \begin{bmatrix} -1 & 0 & 0 & 0 & 0 & 0 & 0 \\ -1 & -1 & -1 & -1 & -1 & 0 & 0 \\ 0 & 0 & 1 & 1 & 1 & 0 & 0 \\ 0 & 0 & 0 & 1 & 1 & 0 & 0 \\ 0 & 0 & 0 & 0 & 1 & 0 & 0 \\ -1 & -1 & -1 & -1 & -1 & -1 & 0 \\ -1 & -1 & -1 & -1 & -1 & -1 & -1 \end{bmatrix}.$

Therefore the equation

$$R.v \quad = \quad M\frac{dX}{dt}$$

gives

$$\begin{bmatrix} 1 & 0 & 1 & 0 & 0 & 0 & 0 & 0 & 0 & 0 & -1 & 0 \\ 0 & 1 & 0 & 1 & 0 & 0 & 0 & 1 & 0 & -1 & 0 & -1 \\ 0 & 0 & 0 & 0 & 1 & 0 & 0 & -1 & 0 & 0 & 0 & 1 \\ 0 & 0 & 0 & 0 & 0 & 1 & 0 & -1 & 0 & 0 & 0 & 1 \\ 0 & 0 & 0 & 0 & 0 & 0 & 1 & -1 & 0 & 0 & 0 & 1 \\ 0 & 0 & 0 & 0 & 0 & 0 & 0 & 0 & 1 & -1 & 0 & 0 \\ 0 & 0 & 0 & 0 & 0 & 0 & 0 & 0 & 0 & 0 & 0 & 0 \end{bmatrix} . \begin{pmatrix} v_1 \\ v_2 \\ v_3 \\ v_4 \\ v_5 \\ v_6 \\ v_7 \\ v_8 \\ v_9 \\ v_{10} \\ v_{11} \\ v_{12} \end{pmatrix} = \begin{bmatrix} -1 & 0 & 0 & 0 & 0 & 0 & 0 \\ -1 & -1 & -1 & -1 & -1 & 0 & 0 \\ 0 & 0 & 1 & 1 & 1 & 0 & 0 \\ 0 & 0 & 0 & 1 & 1 & 0 & 0 \\ 0 & 0 & 0 & 0 & 1 & 0 & 0 \\ -1 & -1 & -1 & -1 & -1 & -1 & 0 \\ -1 & -1 & -1 & -1 & -1 & -1 & -1 \end{bmatrix} . \begin{pmatrix} \dot{x}_1 \\ \dot{x}_2 \\ \dot{x}_3 \\ \dot{x}_4 \\ \dot{x}_5 \\ \dot{x}_6 \\ \dot{x}_7 \end{pmatrix}$$

$$\begin{bmatrix} v_1 & 0 & v_3 & 0 & 0 & 0 & 0 & 0 & 0 & 0 & -v_{11} & 0 \\ 0 & v_2 & 0 & v_4 & 0 & 0 & 0 & v_8 & 0 & -v_{10} & 0 & -v_{12} \\ 0 & 0 & 0 & 0 & v_5 & 0 & 0 & -v_8 & 0 & 0 & 0 & v_{12} \\ 0 & 0 & 0 & 0 & 0 & v_6 & 0 & -v_8 & 0 & 0 & 0 & v_{12} \\ 0 & 0 & 0 & 0 & 0 & 0 & v_7 & -v_8 & 0 & 0 & 0 & v_{12} \\ 0 & 0 & 0 & 0 & 0 & 0 & 0 & 0 & v_9 & -v_{10} & 0 & 0 \\ 0 & 0 & 0 & 0 & 0 & 0 & 0 & 0 & 0 & 0 & 0 & 0 \end{bmatrix} = \begin{bmatrix} -\dot{x}_1 & 0 & 0 & 0 & 0 & 0 & 0 \\ -\dot{x}_1 & -\dot{x}_2 & -\dot{x}_3 & -\dot{x}_4 & -\dot{x}_5 & 0 & 0 \\ 0 & 0 & \dot{x}_3 & \dot{x}_4 & \dot{x}_5 & 0 & 0 \\ 0 & 0 & 0 & \dot{x}_4 & \dot{x}_5 & 0 & 0 \\ 0 & 0 & 0 & 0 & \dot{x}_5 & 0 & 0 \\ -\dot{x}_1 & -\dot{x}_2 & -\dot{x}_3 & -\dot{x}_4 & -\dot{x}_5 & -\dot{x}_6 & 0 \\ -\dot{x}_1 & -\dot{x}_2 & -\dot{x}_3 & -\dot{x}_4 & -\dot{x}_5 & -\dot{x}_6 & -\dot{x}_7 \end{bmatrix}.$$

$$\begin{matrix} \\ \\ \\ \\ \\ \text{Independent Species} \uparrow \\ \text{Dependent Species} \rightarrow \end{matrix} \begin{bmatrix} v_1 & 0 & v_3 & 0 & 0 & 0 & 0 & 0 & 0 & 0 & -v_{11} & 0 \\ 0 & v_2 & 0 & v_4 & 0 & 0 & 0 & v_8 & 0 & -v_{10} & 0 & -v_{12} \\ 0 & 0 & 0 & 0 & v_5 & 0 & 0 & -v_8 & 0 & 0 & 0 & v_{12} \\ 0 & 0 & 0 & 0 & 0 & v_6 & 0 & -v_8 & 0 & 0 & 0 & v_{12} \\ 0 & 0 & 0 & 0 & 0 & 0 & v_7 & -v_8 & 0 & 0 & 0 & v_{12} \\ 0 & 0 & 0 & 0 & 0 & 0 & 0 & 0 & v_9 & -v_{10} & 0 & 0 \\ 0 & 0 & 0 & 0 & 0 & 0 & 0 & 0 & 0 & 0 & 0 & 0 \end{bmatrix} = \begin{bmatrix} -\dot{x}_1 & 0 & 0 & 0 & 0 & 0 & 0 \\ -\dot{x}_1 & -\dot{x}_2 & -\dot{x}_3 & -\dot{x}_4 & -\dot{x}_5 & 0 & 0 \\ 0 & 0 & \dot{x}_3 & \dot{x}_4 & \dot{x}_5 & 0 & 0 \\ 0 & 0 & 0 & \dot{x}_4 & \dot{x}_5 & 0 & 0 \\ 0 & 0 & 0 & 0 & \dot{x}_5 & 0 & 0 \\ -\dot{x}_1 & -\dot{x}_2 & -\dot{x}_3 & -\dot{x}_4 & -\dot{x}_5 & -\dot{x}_6 & 0 \\ -\dot{x}_1 & -\dot{x}_2 & -\dot{x}_3 & -\dot{x}_4 & -\dot{x}_5 & -\dot{x}_6 & -\dot{x}_7 \end{bmatrix}.$$

Multiplying out the lower partition, we obtain

$$V\frac{dX}{dt} = 0,$$
$$\Rightarrow \dot{x}_1 + \dot{x}_2 + \dot{x}_3 + \dot{x}_4 + \dot{x}_5 + \dot{x}_6 + \dot{x}_7 = 0,$$

the independent equations are

$$
\begin{aligned}
-\dot{x}_1 &= v_1 + v_3 - v_{11} \\
-\dot{x}_1 - \dot{x}_2 - \dot{x}_3 - \dot{x}_4 - \dot{x}_5 &= v_2 + v_4 + v_8 - v_{10} - v_{12} \\
\dot{x}_3 + \dot{x}_4 + \dot{x}_5 &= v_5 - v_8 + v_{12} \\
\dot{x}_4 + \dot{x}_5 &= v_6 - v_8 + v_{12} \\
\dot{x}_5 &= v_7 - v_8 + v_{12} \\
-\dot{x}_1 - \dot{x}_2 - \dot{x}_3 - \dot{x}_4 - \dot{x}_5 - \dot{x}_6 &= v_9 - v_{10}
\end{aligned}
$$

Advanced Analysis

$$
S = \begin{bmatrix} S_R \\ S_0 \end{bmatrix} \begin{matrix} \}\text{Independent Species} \\ \}\text{Dependent Species} \end{matrix}
$$

Since the bottom S_0 dependent rows can be derived by linear combinations of the top S_R rows, we can define a matrix, the link matrix(L_0),that can carry out this operation:

$$
\begin{aligned}
S_0 &= L_0 S_R. \\
S &= \begin{bmatrix} S_R \\ S_0 \end{bmatrix} = \begin{bmatrix} I \\ L_0 \end{bmatrix} = L S_R
\end{aligned}
$$

where

$$
L = \begin{bmatrix} I \\ L_0 \end{bmatrix}
$$

We know that

$$
\begin{aligned}
\frac{dX}{dt} &= S.v \\
\Rightarrow \begin{bmatrix} I \\ L_0 \end{bmatrix} S_R v = \frac{dX}{dt} &= \begin{bmatrix} \frac{dX_i}{dt} \\ \frac{dX_d}{dt} \end{bmatrix}
\end{aligned}
$$

where X_i are the independent species and X_d are the dependent species.

$$
S_R v = \frac{dX_i}{dt} \quad (1)
$$

$$
L_0 S_R v = \frac{dX_d}{dt} \quad (2)
$$

Equation (1) multiply by L_0 and subtract by equation (2) then we have

$$
\frac{dX_d}{dt} - L_0 \frac{dX_i}{dt} = 0
$$

Integrating above equation then we get

$$
\begin{aligned}
X_d &= L_0 X_i + T \\
X_d - L_0 X_i &= T \\
\begin{bmatrix} -L_0 & I \end{bmatrix} \begin{bmatrix} X_i \\ X_d \end{bmatrix} &= T
\end{aligned}
$$

where

$$\Gamma = \begin{bmatrix} -L_0 & I \end{bmatrix}$$

and

$$X = (X_i, X_d)$$

According to M value of Γ is $\begin{bmatrix} -1 & -1 & -1 & -1 & -1 & -1 & -1 \end{bmatrix}$ and $X = \begin{pmatrix} x_1 \\ x_2 \\ x_3 \\ x_4 \\ x_5 \\ x_6 \\ x_7 \end{pmatrix}$ then we have

$$\Gamma X = T$$

$$\Rightarrow -x_1 - x_2 - x_3 - x_4 - x_5 - x_6 - x_7 = T$$

$$\Gamma = \begin{bmatrix} -L_0 & I \end{bmatrix}, \quad \begin{bmatrix} -L_0 & I \end{bmatrix} \begin{bmatrix} S_R \\ S_0 \end{bmatrix} = 0$$

$$\searrow \quad \swarrow$$

$$\Gamma S = 0$$

S9 Appendix B: Stability of NH_4^+−Source Systems

Let X_1, X_2 and X_6 be the small perturbation to the steady state concentration $\left(x_1^*, x_2^*, x_6^*\right)$ so that the co-ordinates of NH_4^+−source metabolites and components of its rate of change are $x_1 = x_1^* + X_1, x_2 = x_2^* + X_2, x_6 = x_6^* + X_6$ and $\dot{x}_1 = \dot{X}_1, \dot{x}_2 = \dot{X}_2, \dot{x}_6 = \dot{X}_6$ respectively, where X_1, X_2, X_6 and $\dot{X}_1, \dot{X}_2, \dot{X}_6$ are very small quantities.

Substituting these into the equations, we obtain following new differential equations in the basin of equilibrium point $\left(x_1^*, x_2^*, x_6^*\right)$.

$$\left.\begin{aligned} \dot{X}_1 &= f_1\left(x_1^* + X_1, x_2^* + X_2, x_6^* + X_6\right) \\ \dot{X}_2 &= f_2\left(x_1^* + X_1, x_2^* + X_2, x_6^* + X_6\right) \\ \dot{X}_6 &= f_6\left(x_1^* + X_1, x_2^* + X_2, x_6^* + X_6\right) \end{aligned}\right\} \tag{3}$$

Expanding R.H.S of equation (3) with the help of Taylor's Series about $\left(x_1^*, x_2^*, x_6^*\right)$ and neglecting second and higher order terms, we get

$$\left.\begin{aligned} \dot{X}_1 &= X_1\left(\frac{\partial f_1}{\partial x_1}\right)^* + X_2\left(\frac{\partial f_1}{\partial x_2}\right)^* + X_6\left(\frac{\partial f_1}{\partial x_6}\right)^* \\ \dot{X}_2 &= X_1\left(\frac{\partial f_2}{\partial x_1}\right)^* + X_2\left(\frac{\partial f_2}{\partial x_2}\right)^* + X_6\left(\frac{\partial f_2}{\partial x_6}\right)^* \\ \dot{X}_6 &= X_1\left(\frac{\partial f_6}{\partial x_1}\right)^* + X_2\left(\frac{\partial f_6}{\partial x_2}\right)^* + X_6\left(\frac{\partial f_6}{\partial x_6}\right)^* \end{aligned}\right\} \tag{4}$$

Superscript $'*'$ indicate values are at equilibrium point. Also $f_1\left(x_1^*, x_2^*, x_6^*\right) = f_2\left(x_1^*, x_2^*, x_6^*\right) = f_6\left(x_1^*, x_2^*, x_6^*\right)$. Let us take

$$\left.\begin{aligned} X_1 &= Ae^{\lambda t} \\ X_2 &= Be^{\lambda t} \\ X_6 &= Ce^{\lambda t} \end{aligned}\right\} \tag{5}$$

From equations (4) and (5), we obtain

$$\left.\begin{aligned}\lambda A &= A\left(\frac{\partial f_1}{\partial x_1}\right)^* + B\left(\frac{\partial f_1}{\partial x_2}\right)^* + C\left(\frac{\partial f_1}{\partial x_6}\right)^*\\ \lambda B &= A\left(\frac{\partial f_2}{\partial x_1}\right)^* + B\left(\frac{\partial f_2}{\partial x_2}\right)^* + C\left(\frac{\partial f_2}{\partial x_6}\right)^*\\ \lambda C &= A\left(\frac{\partial f_6}{\partial x_1}\right)^* + B\left(\frac{\partial f_6}{\partial x_2}\right)^* + C\left(\frac{\partial f_6}{\partial x_6}\right)^*\end{aligned}\right\} \tag{6}$$

Which is a system of linear equations. Therefore, for non trivial solution of the system, we have

$$\begin{vmatrix} -\lambda + \left(\frac{\partial f_1}{\partial x_1}\right)^* & \left(\frac{\partial f_1}{\partial x_2}\right)^* & \left(\frac{\partial f_1}{\partial x_6}\right)^* \\ \left(\frac{\partial f_2}{\partial x_1}\right)^* & -\lambda + \left(\frac{\partial f_2}{\partial x_2}\right)^* & \left(\frac{\partial f_2}{\partial x_6}\right)^* \\ \left(\frac{\partial f_6}{\partial x_1}\right)^* & \left(\frac{\partial f_6}{\partial x_2}\right)^* & -\lambda + \left(\frac{\partial f_6}{\partial x_6}\right)^* \end{vmatrix} = 0. \tag{7}$$

Simplifying above determinant, we get cubic equation in λ, which is known as characteristic equation of the system and is given as

$$\lambda^3 + \lambda^2 A^* + \lambda B^* + C^* = 0 \tag{8}$$

Where

$$\begin{aligned} A^* &= -k_1 - k_{11} - k_2 + \frac{k_{12} x_6^*}{\left(1 + x_6^*\right)^2} - \frac{k_{12}}{1 + x_6^*} \\ B^* &= -k_1 k_2 + (k_1 k_{12} + k_{11} k_{12} + k_{12} + k_2)\frac{x_6^*}{\left(1 + x_6^*\right)} - (k_1 k_{12} + k_{11} k_{12} + k_{12} + k_2)\frac{1}{\left(1 + x_6^*\right)} \\ &\& \\ C^* &= -\frac{k_1 k_{12} k_2}{1 + x_6^*} + \frac{k_1 k_{12} k_2 x_6^*}{\left(1 + x_6^*\right)^2} \end{aligned}$$

Now, solving (8) for λ, we get

$$\left.\begin{aligned} \lambda_1 &= \frac{1}{2}\left(-k_1 - k_{11} - k_2 - \sqrt{-4k_1 k_2 + (k_1 + k_{11} + k_2)^2}\right) \\ \lambda_2 &= \frac{1}{2}\left(-k_1 - k_{11} - k_2 + \sqrt{-4k_1 k_2 + (k_1 + k_{11} + k_2)^2}\right) \\ \lambda_6 &= -\frac{k_{12}}{\left(1 + x_6^*\right)} \end{aligned}\right\} \tag{9}$$

From equation (9), it is clear that all the eigenvalues are real and negative. Therefore, the equilibrium point is a stable node or sink i.e., asymptotically stable.

S10 Appendix C: Stability of NH_4^+−Sink Systems

With the assumption that intermediate N−forms hydroxylamine and nitrite transforms faster, the stability of the system is therefore determined by the dynamics of ammonium and nitrate only.

Let X_1 and X_6 be the small perturbation to the steady state concentration $\left(x_1^*, x_6^*\right)$ so that the co-ordinates of NH_4^+−sink metabolites and components of its rate of change are $x_1 = x_1^* + X_1, x_6 =$

$x_6^* + X_6$ and $\dot{x}_1 = \dot{X}_1, \dot{x}_6 = \dot{X}_6$ respectively, where X_1, X_6 and $\dot{X}_1, \dot{X}_6$ are very small quantities.

Substituting these into the equations, we obtain following new differential equations in the basin of equilibrium point $\left(x_1^*, x_6^*\right)$.

$$\left.\begin{aligned}\dot{X}_1 &= f_1\left(x_1^* + X_1, x_6^* + X_6\right)\\ \dot{X}_6 &= f_6\left(x_1^* + X_1, x_6^* + X_6\right)\end{aligned}\right\} \tag{10}$$

Expanding R.H.S of equation (10) with the help of Taylor's Series about $\left(x_1^*, x_6^*\right)$ and neglecting second and higher order terms, we get

$$\left.\begin{aligned}\dot{X}_1 &= X_1\left(\frac{\partial f_1}{\partial x_1}\right)^* + X_6\left(\frac{\partial f_1}{\partial x_6}\right)^*\\ \dot{X}_6 &= X_1\left(\frac{\partial f_6}{\partial x_1}\right)^* + X_6\left(\frac{\partial f_6}{\partial x_6}\right)^*\end{aligned}\right\} \tag{11}$$

Superscript $'*'$ indicate values are at equilibrium point. Also $f_1\left(x_1^*, x_6^*\right) = f_6\left(x_1^*, x_6^*\right)$.
Let us take

$$\left.\begin{aligned}X_1 &= Ae^{\lambda t}\\ X_6 &= Be^{\lambda t}\end{aligned}\right\} \tag{12}$$

From equations (11) and (12), we obtain

$$\left.\begin{aligned}\lambda A &= A\left(\frac{\partial f_1}{\partial x_1}\right)^* + B\left(\frac{\partial f_1}{\partial x_6}\right)^*\\ \lambda B &= A\left(\frac{\partial f_6}{\partial x_1}\right)^* + B\left(\frac{\partial f_6}{\partial x_6}\right)^*\end{aligned}\right\} \tag{13}$$

Which is a system of linear equations. Therefore, for non trivial solution of the system, we have

$$\begin{vmatrix} -\lambda + \left(\frac{\partial f_1}{\partial x_1}\right)^* & \left(\frac{\partial f_1}{\partial x_6}\right)^* \\ \left(\frac{\partial f_6}{\partial x_1}\right)^* & -\lambda + \left(\frac{\partial f_6}{\partial x_6}\right)^* \end{vmatrix} = 0. \tag{14}$$

Simplifying above determinant, we get cubic equation in λ, which is known as characteristic equation of the system and is given as

$$\lambda^2 + \lambda A^* + B^* = 0 \tag{15}$$

Where

$$\begin{aligned} A^* &= \frac{k_{11}^2}{4k_3} - k_{11} + k_3 + \frac{(k_4 - 2k_9)^2}{4k_9} \\ & \& \\ B^* &= \frac{(k_{11} - 2k_3)^2 (k_4 - 2k_9)^2}{16 k_3 k_9} \end{aligned}$$

Now, solving (15) for λ, we get

$$\left.\begin{aligned}\lambda_1 &= -\frac{(k_{11} - 2k_3)^2}{4k_3}\\ \lambda_6 &= -\frac{(k_4 - 2k_9)^2}{4k_9}\end{aligned}\right\} \tag{16}$$

From equation (16), it is clear that all the eigenvalues are real and negative. Therefore, the equilibrium point is a stable node or sink i.e., asymptotically stable.

List of Figures

S1 Nitrogen Biochemical Network 15
S2 Dynamics of Nitrogen Metabolites 16
S3 Transient Dynamics of Ammonium and Nitrate 17
S4 Simulating Responses of NH_4^+ & NO_3^- to External NH_4^+ Inputs (Top-Down Effect) . 18
S5 Simulating Responses of NH_4^+ & NO_3^- to External NH_4^+ Inputs (Bottom-Up Effect) . 19
S6 Schematic Diagram of the Feedback Connected Monotone System 20
S7 Phase Plane Plot 20
S8 Species Graph of Ammonium Source Systems 21
S9 Monotonicty of NH_4^+-Source Systems 21
S10 Transient Dynamics of Ammonium and Nitrate in the NH_4^+-Source Systems 22
S11 Species Graph of Ammonium Sink Systems 23
S12 Monotonicty of NH_4^+-Sink Systems 24
S13 Transient Dynamics of Ammonium and Nitrate in the NH_4^+-Sink Systems 25
S14 Simulating Responses of NH_4^+-Source Systems to External Step Inputs 26
S15 Simulating Responses of NH_4^+-Sink Systems to External Step Inputs 27

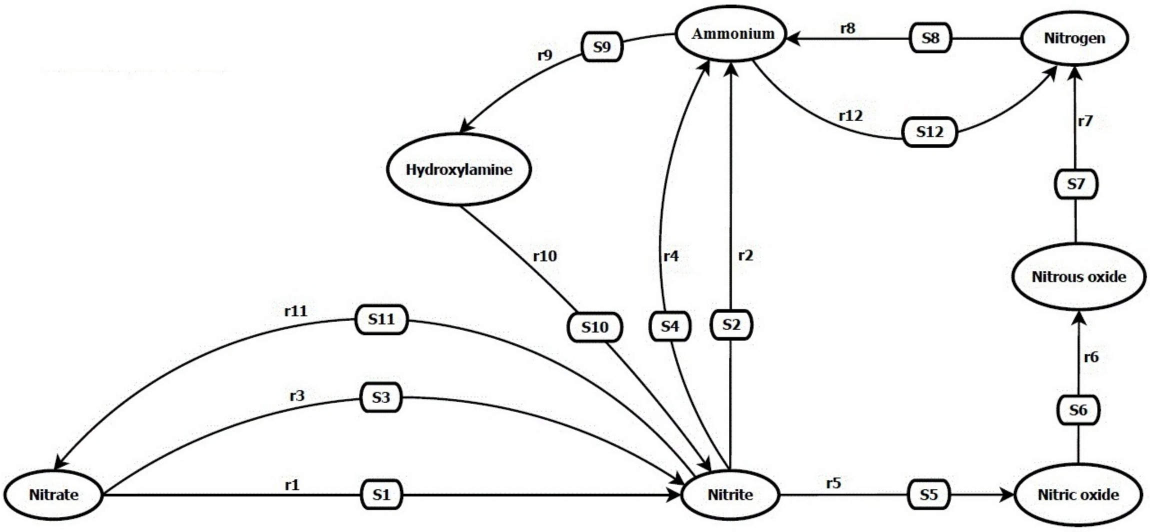

Figure S1: **Nitrogen Biochemical Network**

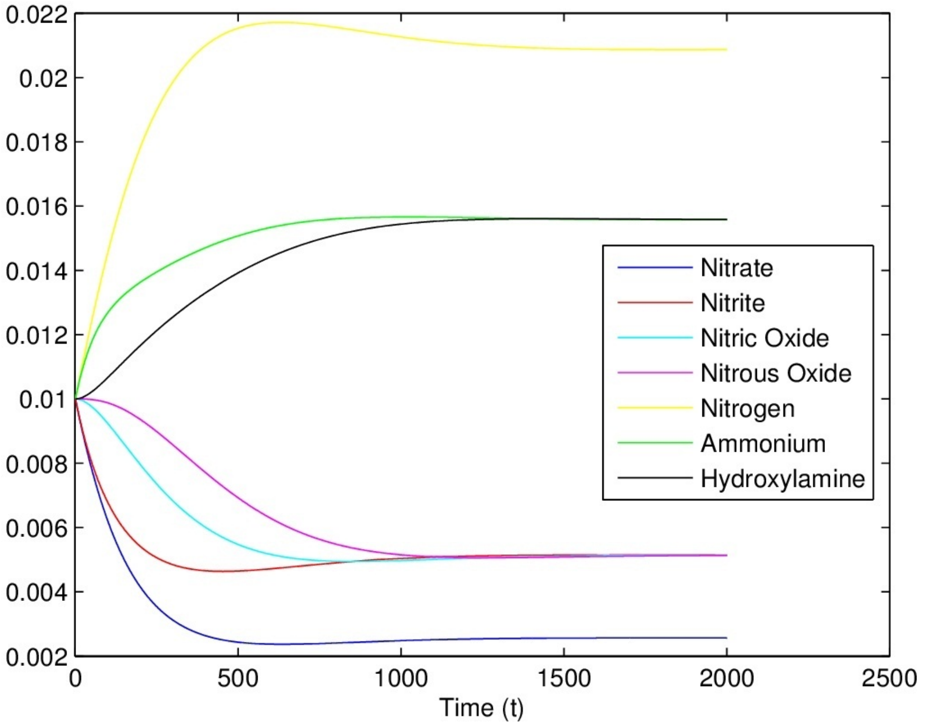

Figure S2: **Dynamics of all the Nitrogen Metabolites: It shows that the system eventually reach to a steady state at which each metabolites maintain its fixed concentration;** $x_1(t)$ **: Nitrate** NO_3^- **(blue),** $x_2(t)$ **: Nitrite** NO_2^- **(red),** $x_3(t)$ **: Nitric Oxide** NO **(cyan),** $x_4(t)$ **: Nitrous Oxide** N_2O **(magenta),** $x_5(t)$ **: Nitrogen** N_2 **(yellow),** $x_6(t)$ **: Ammonium** NH_4^+ **(green),** $x_7(t)$ **: Hydroxylamine** NH_2OH **(black). Numerical simulations have been carried out with the constant reaction coefficient** $k_i = 0.5, i = 1 \ldots 12, k_{m_i} = 1, i = 1 \ldots 12$ **and initial condition** $x_i(0) = 0.01, i = 1 \ldots 7$.

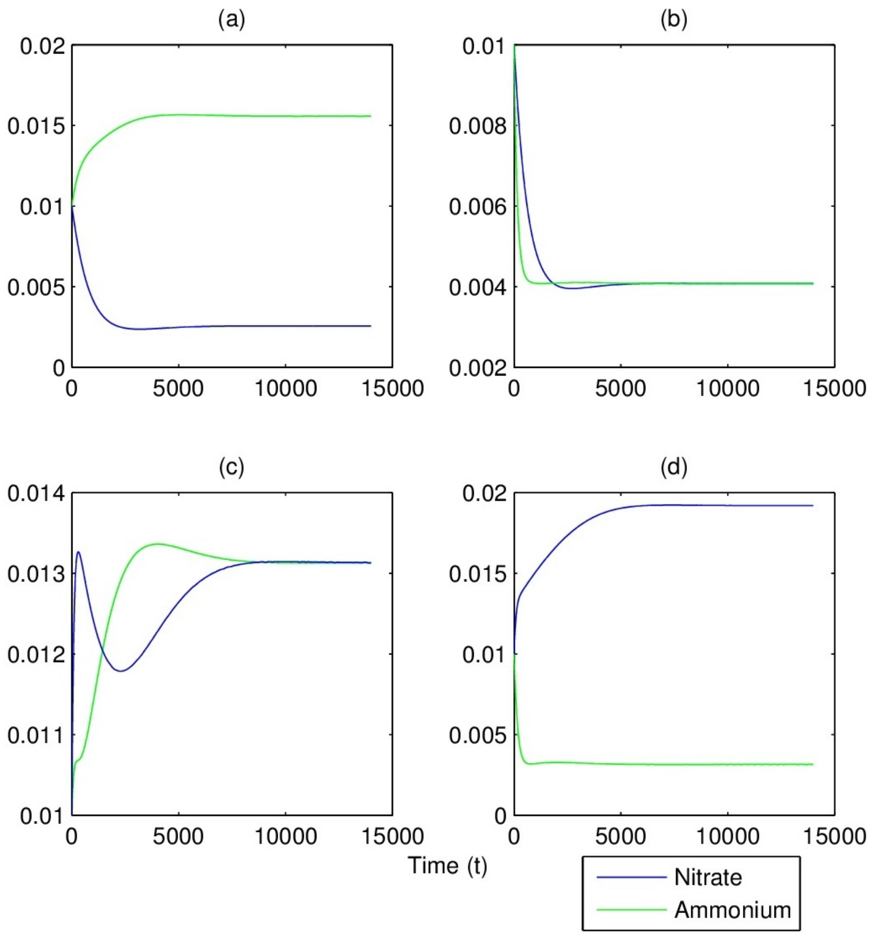

Figure S3: **Transient Dynamics of Ammonium and Nitrate: Changes in nitrification rate lead to opposite trends and steady states of ammonium and nitrate concentrations.**

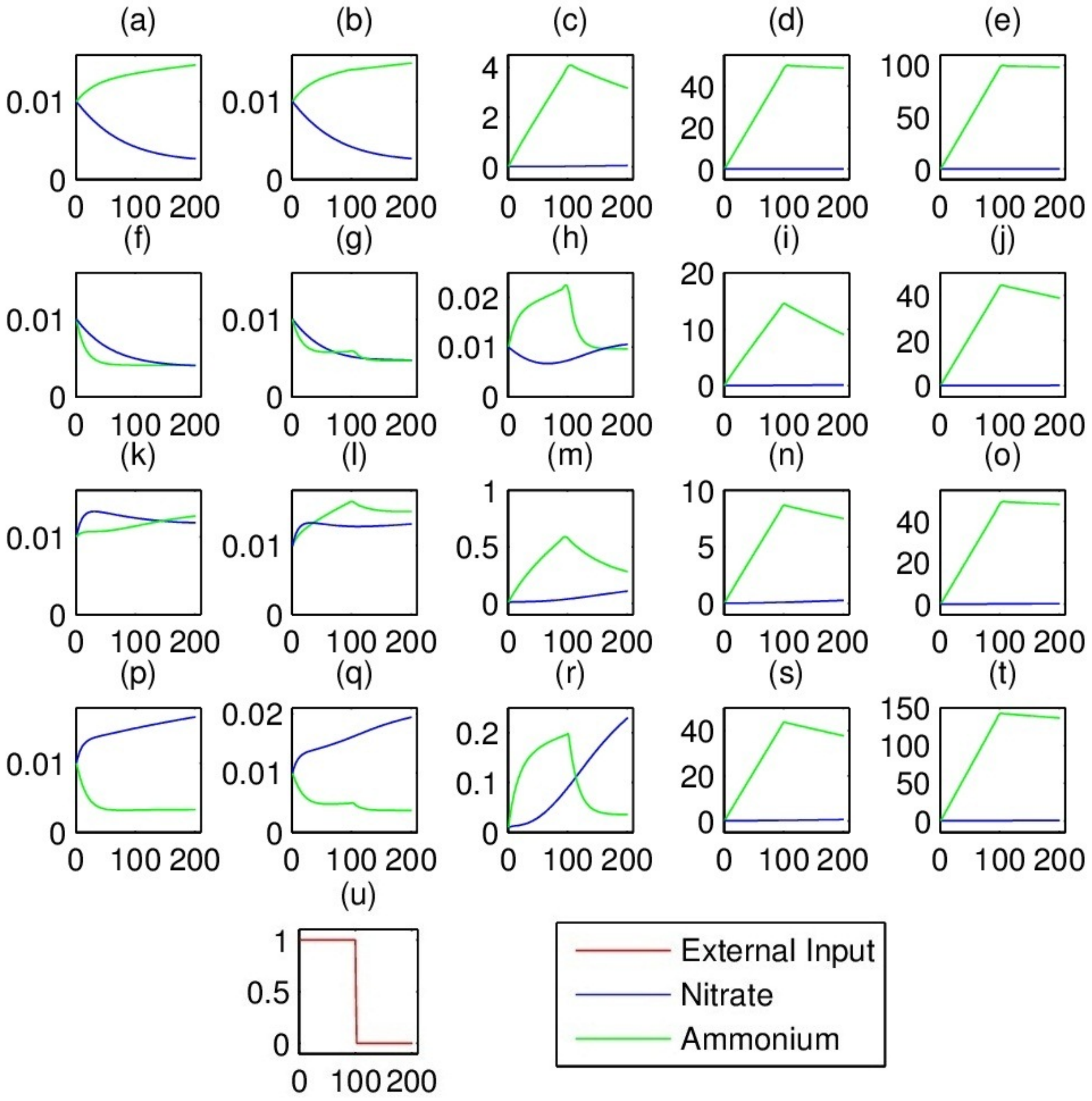

Figure S4: **Responses of the N-Biochemical System to External Ammonium Inputs:** (a) **Top-Down Step Inputs:** $'a'$ **in the figure corresponds to** $'a'$ **in the table 3** (a); $'b'$ **in the figure corresponds to** $'b'$ **in the table** $4a$, **and so on. It shows that elevated ammonium inputs can breakdown the stable functioning of the** $N-$**biochemical system and consequently alter soil availability of ammonium and nitrate (Table S3).**

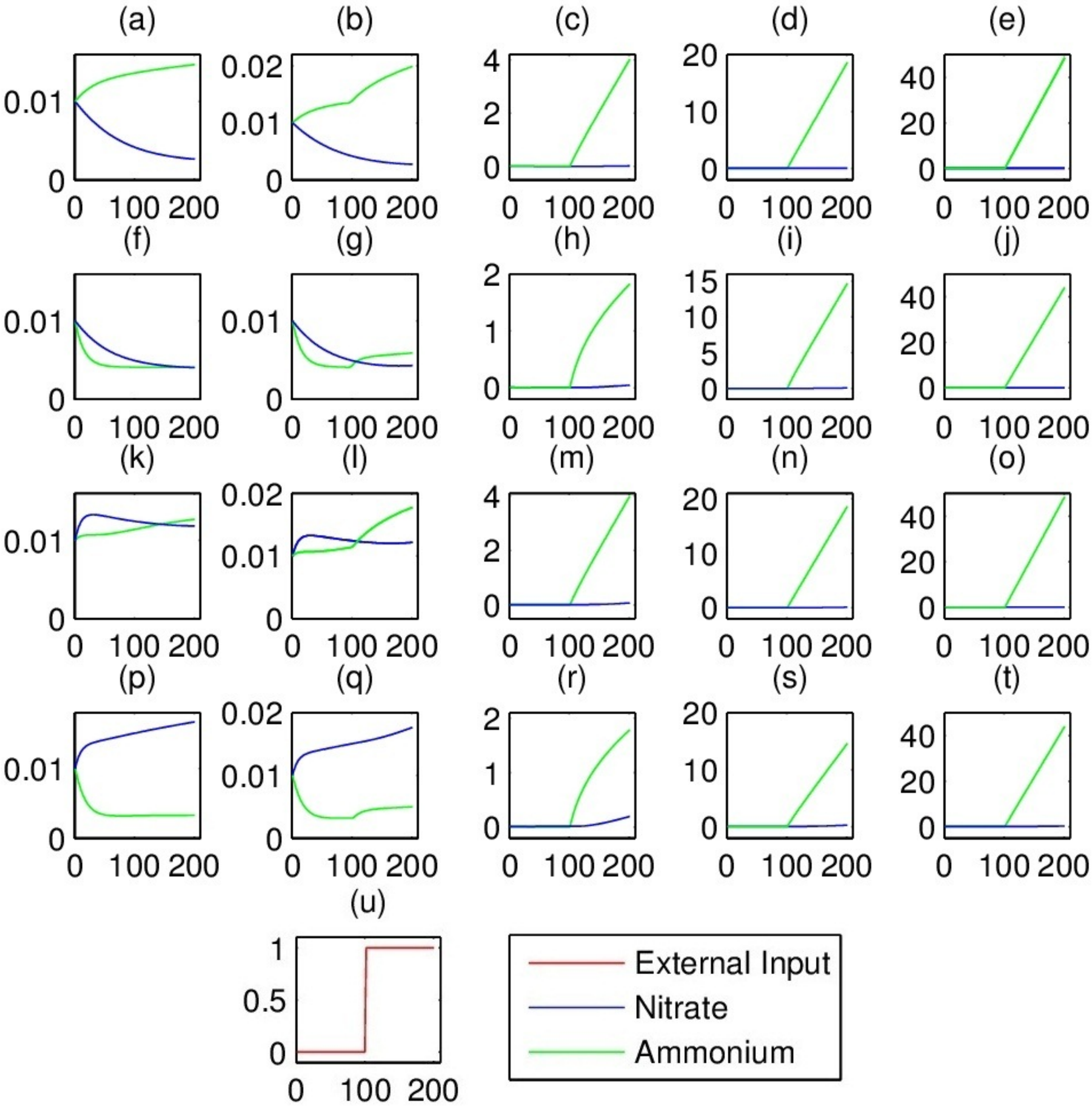

Figure S5: **Responses of the N-Biochemical System to External Ammonium Inputs:** (a) **Bottom-Up Step Inputs:** $'a'$ **in the figure corresponds to** $'a'$ **in the table** $3(b)$; $'b'$ **in the figure corresponds to** $'b'$ **in the table 4b, and so on. It shows that elevated ammonium inputs can breakdown the stable functioning of the** $N-$**biochemical system and consequently alter soil availability of ammonium and nitrate (Table S3).**

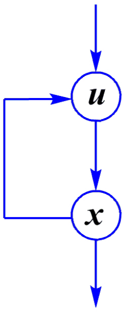

Figure S6: **Schematic Diagram of the Feedback Connected Monotone System.**

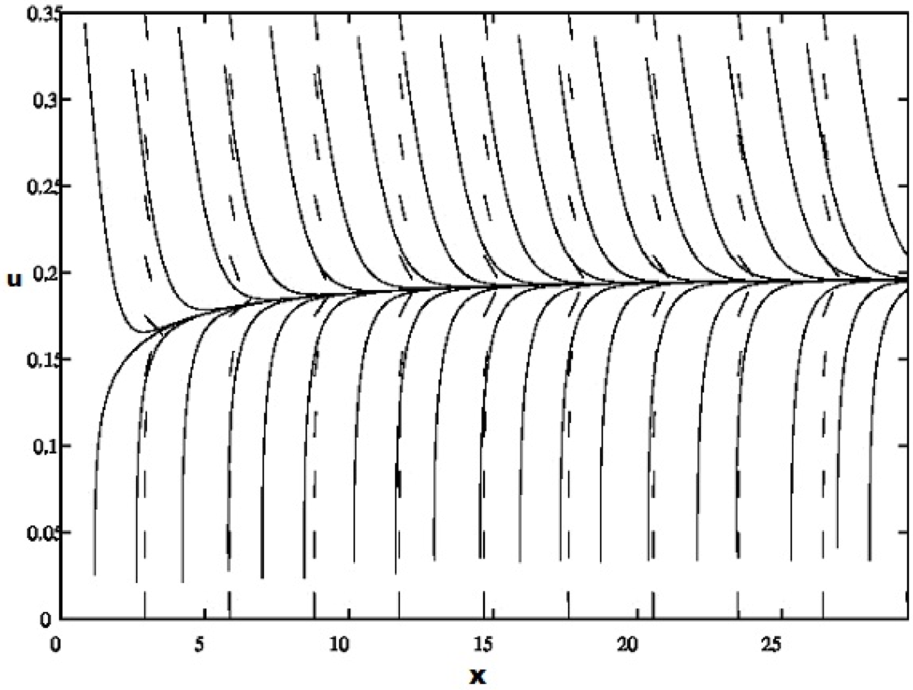

Figure S7: **Phase Plane Plot Showing Monotonicity Behavior of the System, in Which the System Trajectories Slide Along a Continuum of Steady States.**

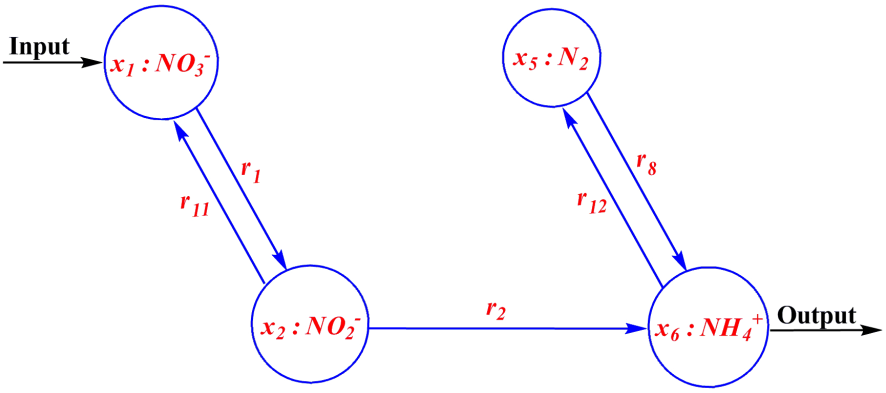

Figure S8: **Species Graph of Ammonium Source Systems**

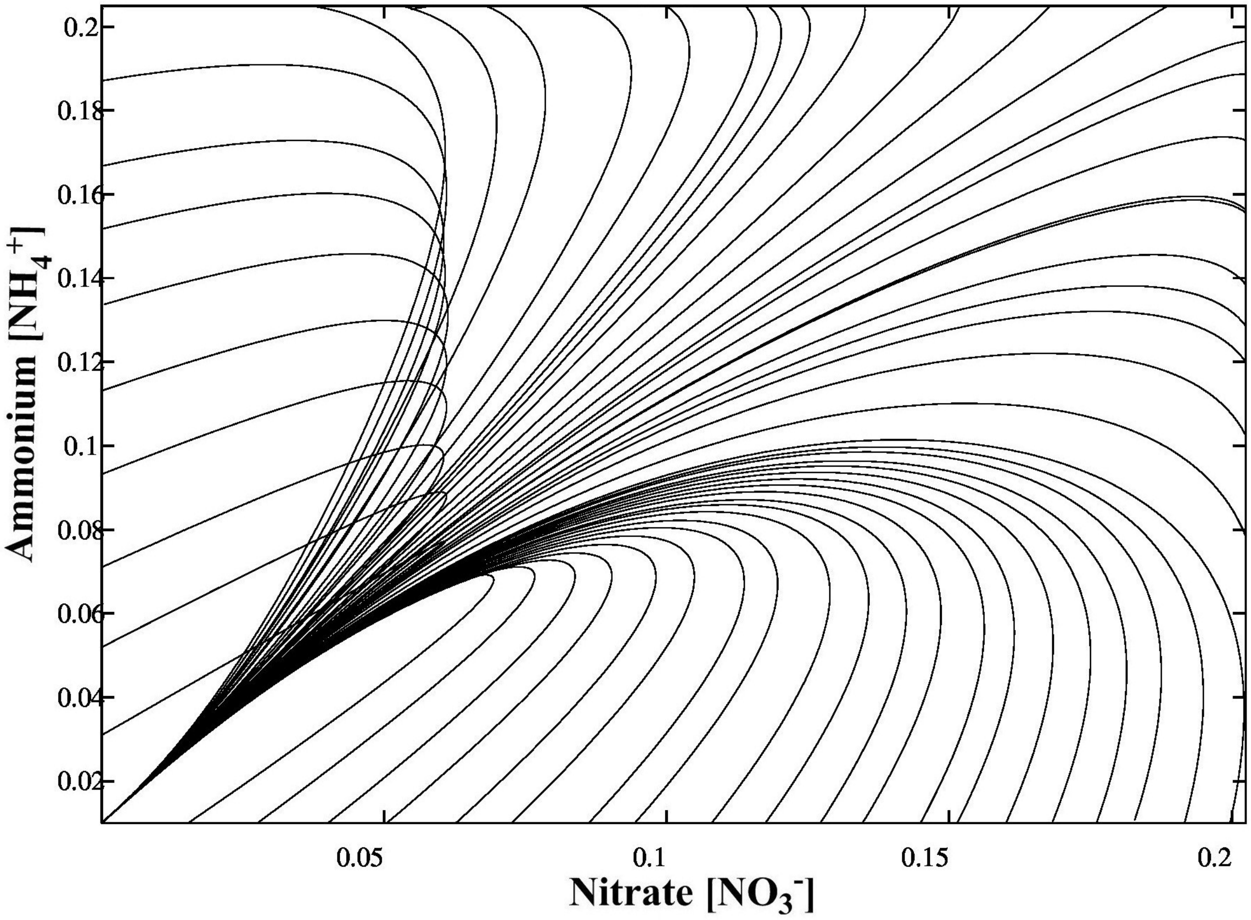

Figure S9: **Montonicity phenomena: it has emerged through dynamic interactions among biochemical pathways in the NH_4^+- source systems. A monotone system responds consistently to perturbations on its components and the system trajectories slide along a continuum of steady states.**

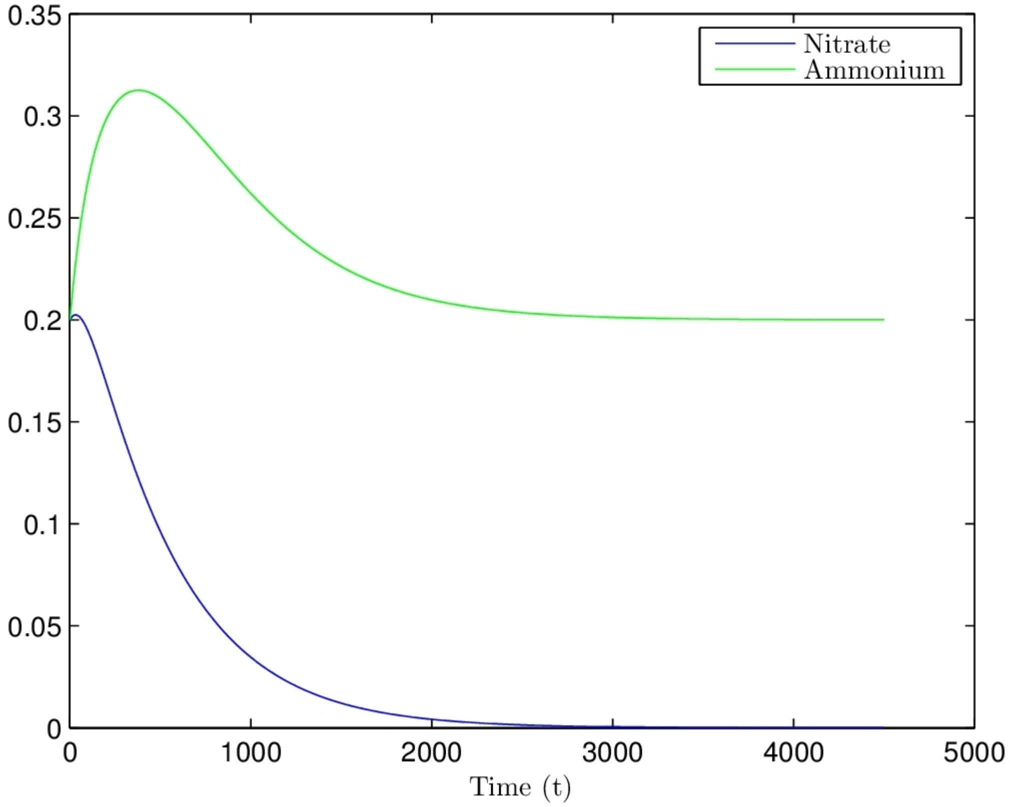

Figure S10: **Transient Dynamics of Ammonium and Nitrate in the NH_4^+−Source Systems: Numerical simulation has been carried out with the parameters** $k_1 = 0.6, k_2 = 0.6, k_8 = 0.5, k_{11} = 0.7, k_{12} = 0.5, k_{m_i} = 1, i = 1, 2, 8, 11, 12$ **and initial condition** $x_i(0) = 0.2, i = 1, 2, 5, 6$.

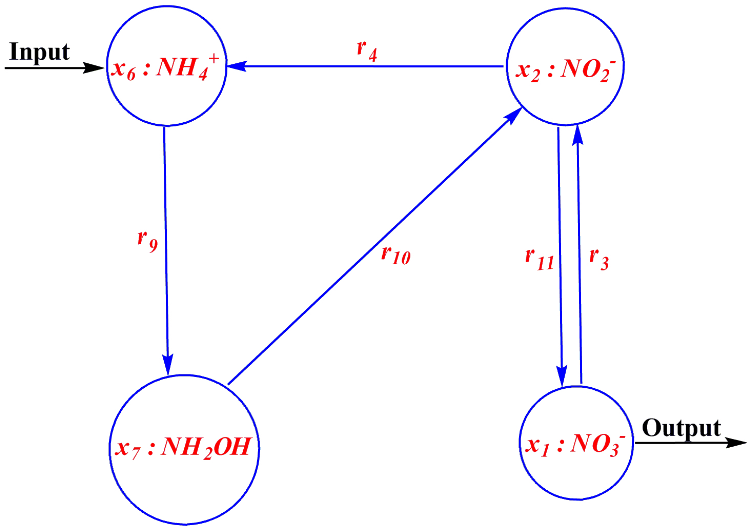

Figure S11: **Species Graph of Ammonium Sink Systems**

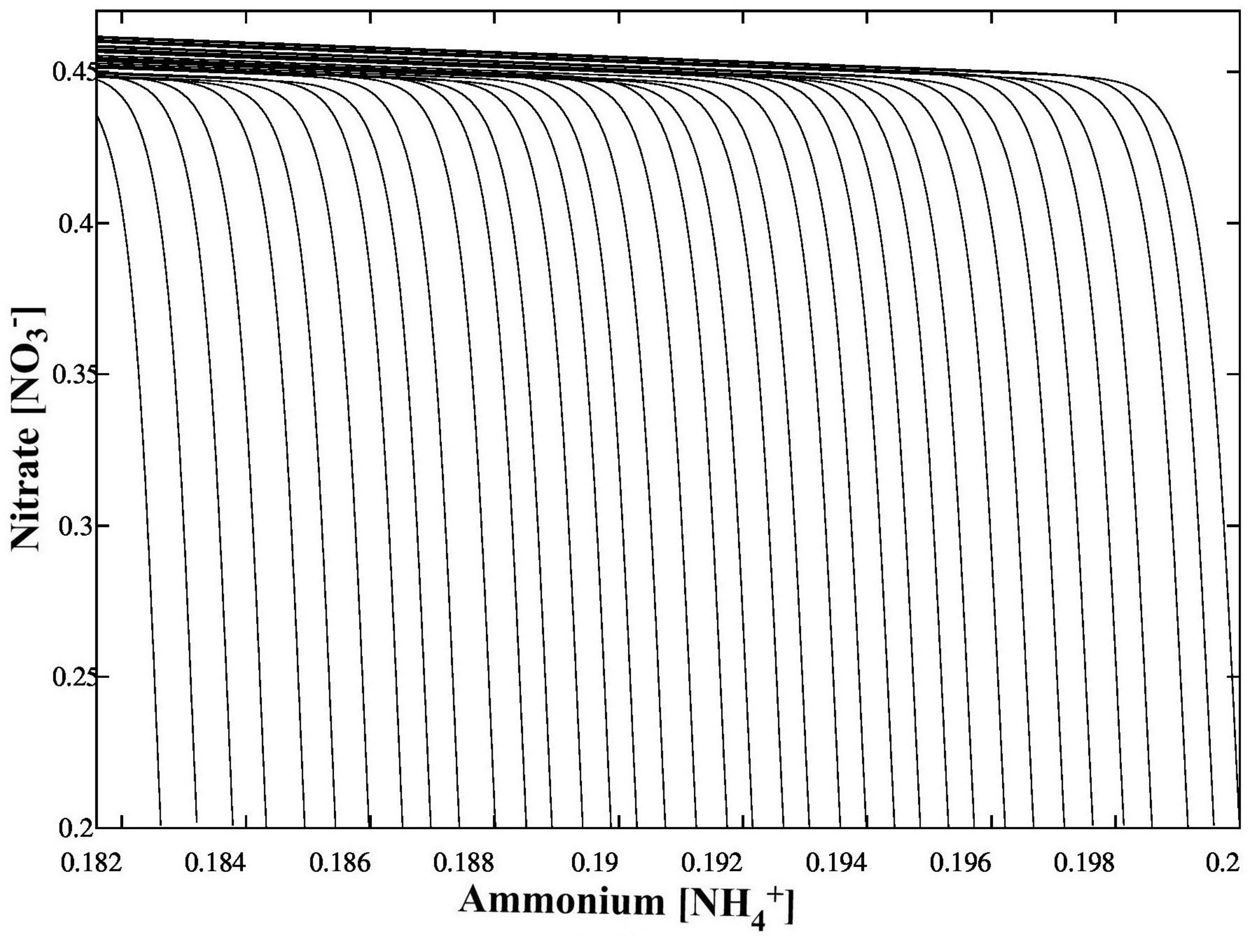

Figure S12: **Montonicity phenomena: it has emerged through dynamic interactions among biochemical pathways in the NH_4^+ – sink systems. A monotone system responds consistently to perturbations on its components and the system trajectories slide along a continuum of steady states.**

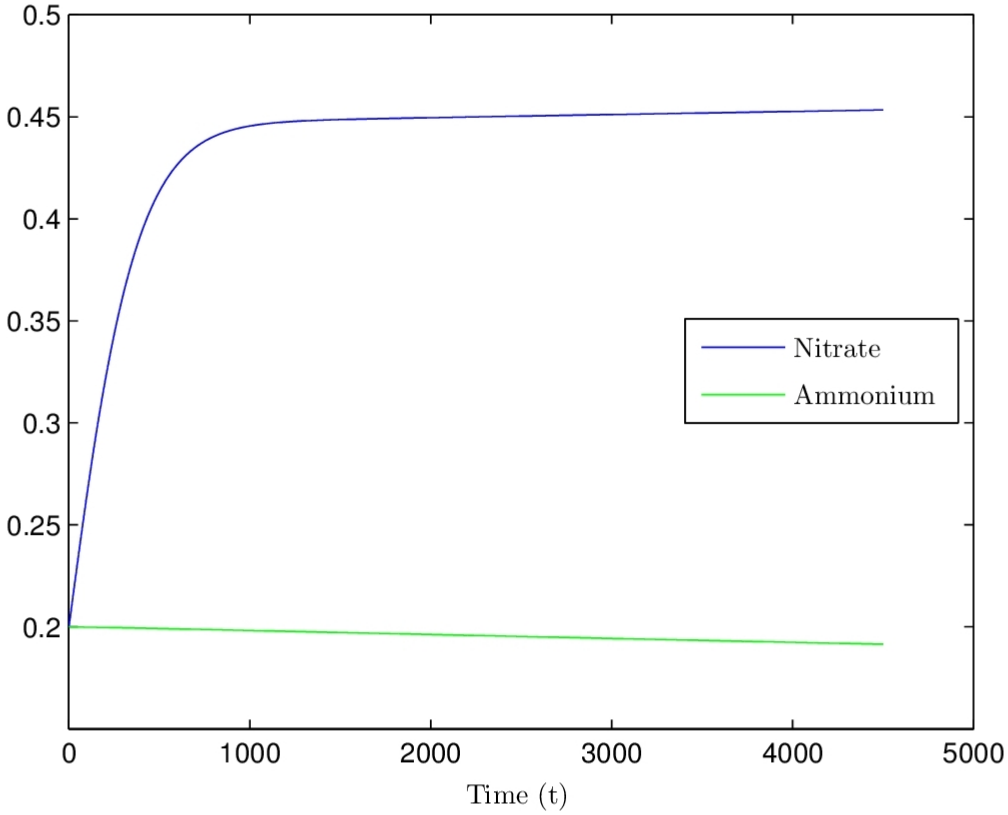

Figure S13: **Transient Dynamics of Ammonium and Nitrate in the** NH_4^+**−Sink Systems: Numerical simulation has been carried out with the parameters** $k_3 = 0.3, k_4 = 0.001, k_9 = 0.002, k_{10} = 0.6, k_{11} = 0.7, k_{m_i} = 1, i = 3,4,9,10,11$ **and initial condition** $x_i(0) = 0.2, i = 1,2,6,7$.

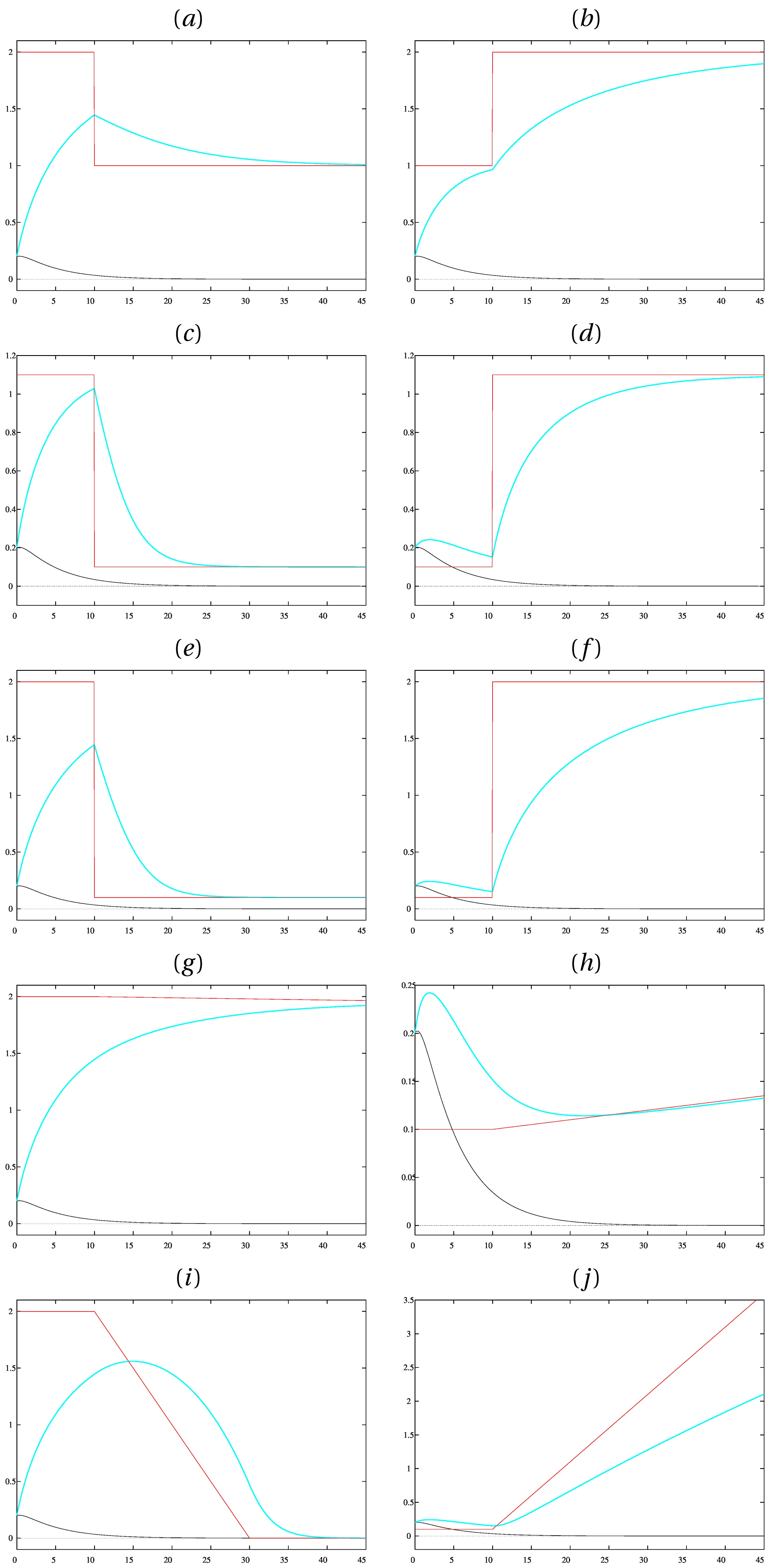

Figure S14: **Simulating Responses of NH_4^+−Source Systems to External Step Inputs: It has been carried out with the parameters $k_1 = 0.6, k_2 = 0.6, k_8 = 0.5, k_{11} = 0.7, k_{12} = 0.5$ and initial condition $x_i(0) = 0.2, i = 1,2,6$; red line: step input, cyan line: Ammonium, black line: Nitrate.**

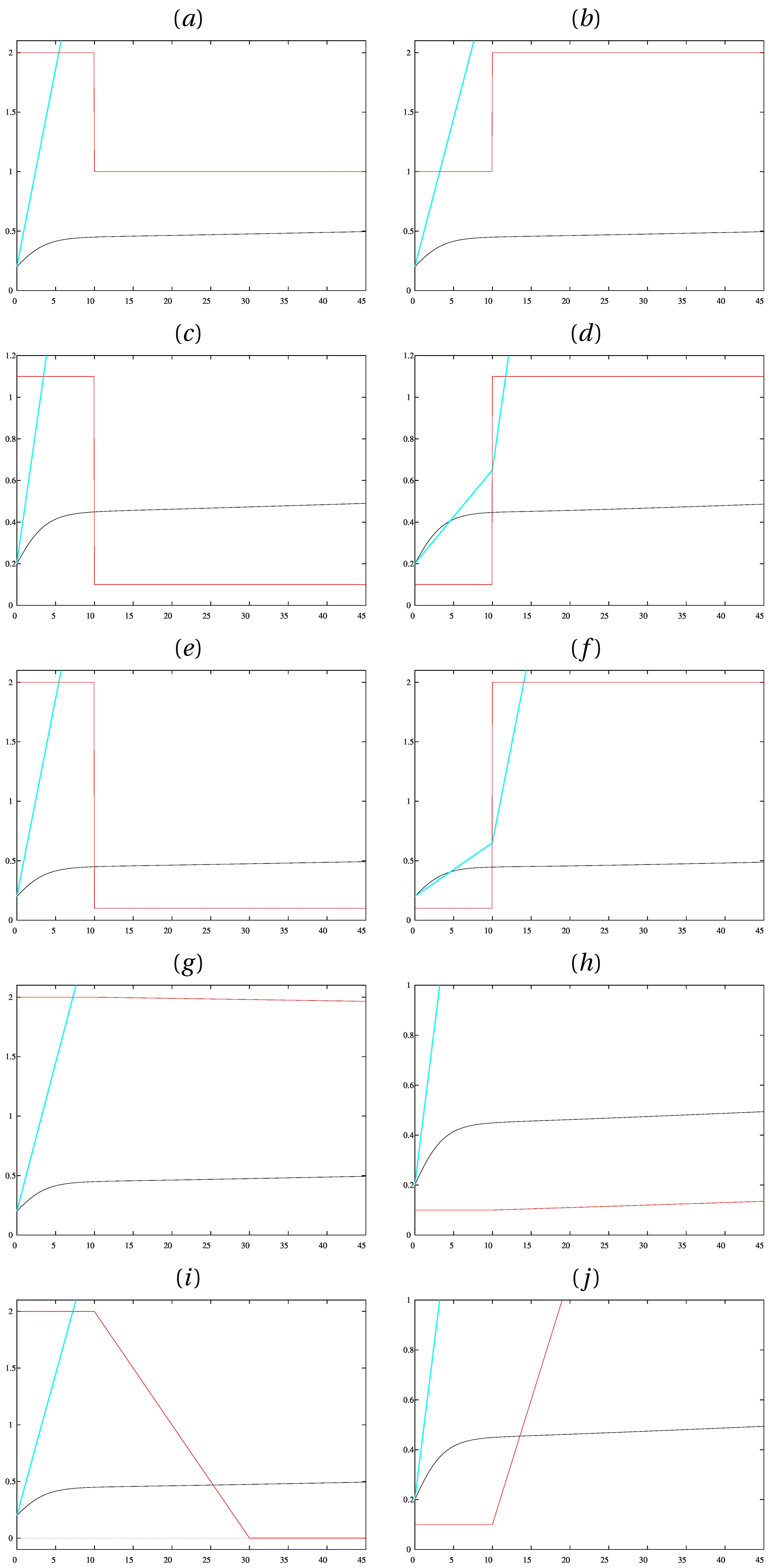

Figure S15: **Simulating Responses of NH_4^+−Sink Systems to External Step Inputs: It has been carried out with the parameters** $k_3 = 0.3, k_4 = 0.001, k_9 = 0.002, k_{10} = 0.6, k_{11} = 0.7$ **and initial condition** $x_i(0) = 0.2, i = 1, 2, 6, 7$; **red line: step input, cyan line: Ammonium, black line: Nitrate.**

List of Tables

S1 Biogeochemical Processes and Associated KEGG Pathways 29
S2 Random Variation of Michaelis Constant k_m in the $N-$Biochemical System 30
S3 Response of Ammonium and Nitrate to External Inputs of Ammonium 31
S4 Simulating Responses of NH_4^+-Source Systems to External Step Inputs 32
S5 Simulating Responses of NH_4^+-Sink Systems to External Step Inputs 33

Table S1: **Biogeochemical Processes and Associated KEGG Pathways**

Nitrogen Biogeochemical Processes	Genes Encoding Enzymes with the KEGG IDs and EC numbers	Nitrogen Transformation Pathways	Reaction Rate Symbols	Biological Structure Associated with the Biochemical Pathways
**Dissimilatory Nitrate Reduction to Ammonia (DNRA)**	K00370 *narG;* nitrate reductase alpha subunit [EC:1.7.99.4]<br>K00371 *narH;* nitrate reductase beta subunit [EC:1.7.99.4]<br>K00374 *narI;* nitrate reductase gamma subunit [EC:1.7.99.4]<br>K00373 *narJ;* nitrate reductase delta subunit<br>K02567 *napA;* periplasmic nitrate reductase NapA [EC:1.7.99.4]<br>K02568 *napB;* cytochrome c-type protein napB	$NO_3^- \longrightarrow NO_2^-$	r_1	s_1 : *narGHIJ, napAB*
	K00362 *nirB;* nitrite reductase (NAD(P)H) large subunit [EC:1.7.1.4]<br>K00363 *nirD;* nitrite reductase (NAD(P)H) small subunit [EC:1.7.1.4]<br>K03385 *nrfA;* cytochrome c-552 [EC:1.7.2.2]<br>K15876 *nrfH;* cytochrome c nitrite reductase small subunit	$NO_2^- \longrightarrow NH_4^+$	r_2	s_2 : *nirBD, nrfAH*
**Assimilatory Nitrate Reduction**	K00367 *narB;* ferredoxin-nitrate reductase [EC:1.7.7.2]<br>K00372 *nasA;* assimilatory nitrate reductase catalytic subunit [EC:1.7.99.4]<br>K00360 *nasB;* assimilatory nitrate reductase electron transfer subunit [EC:1.7.99.4]	$NO_3^- \longrightarrow NO_2^-$	r_3	s_3 : *narB, nasAB*
	K00366 *nirA;* ferredoxin-nitrite reductase [EC:1.7.7.1]	$NO_2^- \longrightarrow NH_4^+$	r_4	s_4 : *nirA,*
**Denitrification**	K00370 *narG;* nitrate reductase alpha subunit [EC:1.7.99.4]<br>K00371 *narH;* nitrate reductase beta subunit [EC:1.7.99.4]<br>K00374 *narI;* nitrate reductase gamma subunit [EC:1.7.99.4]<br>K00373 *narJ;* nitrate reductase delta subunit<br>K02567 *napA;* periplasmic nitrate reductase NapA [EC:1.7.99.4]s<br>K02568 *napB;* cytochrome c-type protein NapB	$NO_3^- \longrightarrow NO_2^-$	r_1	s_1 : *narGHIJ, napAB*
	K00368 *nirK;* nitrite reductase (NO-forming) [EC:1.7.2.1]<br>K15864 *nirS;* nitrite reductase (NO-forming) / hydroxylamine reductase [EC:1.7.2.1 1.7.99.1]	$NO_2^- \longrightarrow NO$	r_5	s_5 : *nirK, nirS*
	K04561 *norB;* nitric oxide reductase subunit B [EC:1.7.2.5]<br>K02305 *norC;* nitric oxide reductase subunit C	$NO \longrightarrow N_2O$	r_6	s_6 : *norBC*
	K00376 *nosZ;* nitrous-oxide reductase [EC:1.7.2.4]	$N_2O \longrightarrow N_2$	r_7	s_7 : *nosZ*
**Nitrogen Fixation**	K02588 *nifH;* nitrogenase iron protein NifH [EC:1.18.6.1]<br>K02586 *nifD;* nitrogenase molybdenum-iron protein alpha chain [EC:1.18.6.1]<br>K02591 *nifK;* nitrogenase molybdenum-iron protein beta chain [EC:1.18.6.1]<br>K00531 *anfG;* nitrogenase delta subunit [EC:1.18.6.1]	$N_2 \longrightarrow NH_4^+$	r_8	s_8 : *nifDKH, anfG*
**Nitrification**	K10944 *amoA;* ammonia monooxygenase subunit A [EC:1.14.18.3 1.14.99.39]<br>K10945 *amoB;* ammonia monooxygenase subunit B<br>K10946 *amoC;* ammonia monooxygenase subunit C	$NH_4^+ \longrightarrow NH_2OH$	r_9	s_9 : *amoABC*
	K10535 *hao;* hydroxylamine dehydrogenase [EC:1.7.2.6]	$NH_2OH \longrightarrow NO_2^-$	r_{10}	s_{10} : *hao*
	K00370 *narG;* nitrate reductase alpha subunit [EC:1.7.99.4]<br>K00371 *narH;* nitrate reductase beta subunit [EC:1.7.99.4]	$NO_2^- \longrightarrow NO_3^-$	r_{11}	s_{11} : *nor*
**Anammox**	⋯ *hzo;* hydrazine oxidoreductase*	$NH_4^+ \longrightarrow N_2$	r_{12}	s_{12} : *hzo*

* KEGG ID and EC number is not assigned in the KEGG database.

Table S2: **Random Variation of Michaelis Constant k_m in the $N-$Biochemical System**

**S. No.**	k_m **Value**	**Time taken to reach at the steady state**	**Plot line style**	**Steady State of $N-$Biochemical System**
(i)	1	13.48	————————	$x_1^* = 0.0026, x_2^* = 0.0051, x_3^* = 0.0051, x_4^* = 0.0051, x_5^* = 0.0209, x_6^* = 0.0156, x_7^* = 0.0156$
(ii)	1+ rand(1)	23.13	- - - - - - - - - -	$x_1^* = 0.0026, x_2^* = 0.0051, x_3^* = 0.0051, x_4^* = 0.0051, x_5^* = 0.0208, x_6^* = 0.0156, x_7^* = 0.0156$
(iii)	2+ rand(1)	45.22	....	$x_1^* = 0.0026, x_2^* = 0.0052, x_3^* = 0.0052, x_4^* = 0.0052, x_5^* = 0.0208, x_6^* = 0.0156, x_7^* = 0.0156$
(iv)	3+ rand(1)	60.43	-.-.-.-.-.-.-.-.-.	$x_1^* = 0.0026, x_2^* = 0.0052, x_3^* = 0.0052, x_4^* = 0.0052, x_5^* = 0.0208, x_6^* = 0.0156, x_7^* = 0.0156$
(v)	4+ rand(1)	77.37	....	$x_1^* = 0.0026, x_2^* = 0.0052, x_3^* = 0.0052, x_4^* = 0.0052, x_5^* = 0.0208, x_6^* = 0.0156, x_7^* = 0.0156$
(vi)	5+ rand(1)	91.12	--.--.--.--.--.--.	$x_1^* = 0.0026, x_2^* = 0.0052, x_3^* = 0.0052, x_4^* = 0.0052, x_5^* = 0.0208, x_6^* = 0.0156, x_7^* = 0.0156$

Table S3: **Response of Ammonium and Nitrate to External Inputs of Ammonium for a Fixed Period Starting from Very Beginning of the Evolution of $N-$Biochemical System**

(a): Top-Down Effect of the Ammonium Step Input

**Dynamic Regimes***	**$x_6: NH_4^+$ & $x_1: NO_3^-$ Level Without External Input**	**Qualitative and Quantitative Changes in NH_4^+ (green) & NO_3^- (blue) Levels With External Input Level k of NH_4^+**							
I<br>Ammonium Rich Regime<br>($k_i = 0.1; i = 1,2,\ldots,12$)	(a)	(b)		(c)		(d)		(e)	
	$x_1 = 0.0026$<br>$x_6 = 0.0147$	$k = 0.0001$		$k = 0.5$		$k = 5$		$k = 10$	
		Change Levels	Dynamic Regimes	Change Levels	Dynamic Regimes	Change Levels	Dynamic Regimes	Change Levels	Dynamic Regimes
		$x_1 = 0.0027$<br>$x_6 = 0.0150$	I	$x_1 = 0.0504$<br>$x_6 = 3.1632$	I	$x_1 = 0.0633$<br>$x_6 = 48.688$	I	$x_1 = 0.0645$<br>$x_6 = 98.2246$	I
II<br>Nitrogen Poor Regime<br>$k_i = 0.1; k_9 = 0.6$	(f)	(g)		(h)		(i)		(j)	
	$x_1 = 0.0040$<br>$x_6 = 0.0041$	$k = 0.001$		$k = 0.01$		$k = 2$		$k = 5$	
		Change Levels	Dynamic Regimes	Change Levels	Dynamic Regimes	Change Levels	Dynamic Regimes	Change Levels	Dynamic Regimes
		$x_1 = 0.0047$<br>$x_6 = 0.0047$	II	$x_1 = 0.0106$<br>$x_6 = 0.0097$	III	$x_1 = 0.1204$<br>$x_6 = 9.0185$	I	$x_1 = 0.1234$<br>$x_6 = 38.6803$	I
III<br>Nearly Identical NH_4^+ and NO_3^- Levels<br>$k_i = 0.1, k_{11} = 0.6$	(k)	(l)		(m)		(n)		(o)	
	$x_1 = 0.0118$<br>$x_6 = 0.0127$	$k = 0.001$		$k = 0.1$		$k = 1$		$k = 5$	
		Change Levels	Dynamic Regimes	Change Levels	Dynamic Regimes	Change Levels	Dynamic Regimes	Change Levels	Dynamic Regimes
		$x_1 = 0.0131$<br>$x_6 = 0.0150$	III	$x_1 = 0.1077$<br>$x_6 = 0.2798$	I	$x_1 = 0.2528$<br>$x_6 = 7.4910$	I	$x_1 = 0.2873$<br>$x_6 = 48.1951$	I
IV<br>Nitrate Rich Regime<br>$k_i = 0.1, k_9 = 0.6, k_{11} = 0.6$	(p)	(q)		(r)		(s)		(t)	
	$x_1 = 0.0167$<br>$x_6 = 0.0033$	$k = 0.001$		$k = 0.1$		$k = 5$		$k = 15$	
		Change Levels	Dynamic Regimes	Change Levels	Dynamic Regimes	Change Levels	Dynamic Regimes	Change Levels	Dynamic Regimes
		$x_1 = 0.0194$<br>$x_6 = 0.0037$	IV	$x_1 = 0.2299$<br>$x_6 = 0.0355$	IV	$x_1 = 0.6051$<br>$x_6 = 37.634$	I	$x_1 = 0.6156$<br>$x_6 = 136.5174$	I

(b): Bottom-Up Effect of the Ammonium Step Input

**Dynamic Regimes***	**$x_6: NH_4^+$ & $x_1: NO_3^-$ Level Without External Input**	**Qualitative and Quantitative Changes in NH_4^+ (green) & NO_3^- (blue) Levels With External Input Level k of NH_4^+**							
I<br>Ammonium Rich Regime<br>($k_i = 0.1; i = 1,2,\ldots,12$)	(a)	(b)		(c)		(d)		(e)	
	$x_1 = 0.0026$<br>$x_6 = 0.0147$	$k = 0.001$		$k = 0.5$		$k = 2$		$k = 5$	
		Change Levels	Dynamic Regimes	Change Levels	Dynamic Regimes	Change Levels	Dynamic Regimes	Change Levels	Dynamic Regimes
		$x_1 = 0.0027$<br>$x_6 = 0.0199$	I	$x_1 = 0.0178$<br>$x_6 = 4.0182$	I	$x_1 = 0.0239$<br>$x_6 = 18.6385$	I	$x_1 = 0.0262$<br>$x_6 = 48.5147$	I
II<br>Nitrogen Poor Regime<br>$k_i = 0.1; k_9 = 0.6$	(f)	(g)		(h)		(i)		(j)	
	$x_1 = 0.0040$<br>$x_6 = 0.0041$	$k = 0.001$		$k = 0.5$		$k = 2$		$k = 5$	
		Change Levels	Dynamic Regimes	Change Levels	Dynamic Regimes	Change Levels	Dynamic Regimes	Change Levels	Dynamic Regimes
		$x_1 = 0.0043$<br>$x_6 = 0.0059$	II	$x_1 = 0.0466$<br>$x_6 = 1.8223$	I	$x_1 = 0.0594$<br>$x_6 = 14.6753$	I	$x_1 = 0.0631$<br>$x_6 = 44.066$	I
III<br>Nearly Identical NH_4^+ and NO_3^- Levels<br>$k_i = 0.1, k_{11} = 0.6$	(k)	(l)		(m)		(n)		(o)	
	$x_1 = 0.0118$<br>$x_6 = 0.0127$	$k = 0.001$		$k = 0.5$		$k = 2$		$k = 5$	
		Change Levels	Dynamic Regimes	Change Levels	Dynamic Regimes	Change Levels	Dynamic Regimes	Change Levels	Dynamic Regimes
		$x_1 = 0.0122$<br>$x_6 = 0.0178$	II	$x_1 = 0.0742$<br>$x_6 = 3.9999$	I	$x_1 = 0.0995$<br>$x_6 = 18.6114$	I	$x_1 = 0.1088$<br>$x_6 = 48.4836$	I
IV<br>Nitrate Rich Regime<br>$k_i = 0.1, k_9 = 0.6, k_{11} = 0.6$	(p)	(q)		(r)		(s)		(t)	
	$x_1 = 0.0167$<br>$x_6 = 0.0033$	$k = 0.001$		$k = 0.5$		$k = 2$		$k = 5$	
		Change Levels	Dynamic Regimes	Change Levels	Dynamic Regimes	Change Levels	Dynamic Regimes	Change Levels	Dynamic Regimes
		$x_1 = 0.0177$<br>$x_6 = 0.0050$	IV	$x_1 = 0.1969$<br>$x_6 = 1.7850$	I	$x_1 = 0.2534$<br>$x_6 = 14.6107$	I	$x_1 = 0.2701$<br>$x_6 = 43.9939$	I

(Initial condition $x_i(0) = 0.01, i = 1,\ldots,7$ and time $t = 20$)

* I: ammonium concentration is more than the 5 times of nitrate concentration (i.e., ammonia rich regimes); II: low availability of both ammonium and nitrate (i.e., nitrogen poor regimes); III: nearly identical concentration of ammonium and nitrate,; IV: nitrate concentration is more than the 5 times of ammonium concentration (i.e., nitrate rich regimes).

Table S4: **Simulating Responses of NH_4^+−Source Systems to External Step Inputs**

Plot No.	Step Input (Red)		Output		
	Upper Level	Lower Level	x_1 (Black)	x_6 (Cyan)	$\frac{x_6}{x_1}$
**Top-Down**					
(a)	2	1	At time $t = 10$, $x_1 = 0.034605$<br>At time $t = 45$ $x_1 = 2.02039e^{-005}$	At time $t = 10$, $x_6 = 1.4458$<br>At time $t = 45$, $x_6 = 1.00893$	At time $t = 10$, $\frac{x_6}{x_1} = 41.7802$<br>At time $t = 45$, $\frac{x_6}{x_1} = 49937.5$
(c)	1.1	0.1	At time $t = 10$, $x_1 = 0.034605$<br>At time $t = 45$, $x_1 = 2.02039e^{-005}$	At time $t = 10$, $x_6 = 1.0269$<br>At time $t = 45$, $x_6 = 0.100035$	At time $t = 10$, $\frac{x_6}{x_1} = 29.6749$<br>At time $t = 45$, $\frac{x_6}{x_1} = 4951.25$
(e)	2	0.1	At time $t = 10$, $x_1 = 0.034605$<br>At time $t = 45$, $x_1 = 2.02039e^{-005}$	At time $t = 10$, $x_6 = 1.4441$<br>At time $t = 45$, $x_6 = 0.100036$	At time $t = 10$, $\frac{x_6}{x_1} = 41.731$<br>At time $t = 45$, $\frac{x_6}{x_1} = 4951.32$
(g) With Slop 1000	2	$\cdots$	At time $t = 10$, $x_1 = 0.034605$<br>At time $t = 45$, $x_1 = 2.02039e^{-005}$	At time $t = 10$, $x_6 = 1.4465$<br>At time $t = 45$, $x_6 = 1.92191$	At time $t = 10$, $\frac{x_6}{x_1} = 41.8003$<br>At time $t = 45$, $\frac{x_6}{x_1} = 95125.8$
(i) With Slop 10	2	$\cdots$	At time $t = 10$, $x_1 = 0.034605$<br>At time $t = 45$, $x_1 = 2.02039e^{-005}$	At time $t = 10$, $x_6 = 1.4465$<br>At time $t = 45$, $x_6 = 0.00044031$	At time $t = 10$, $\frac{x_6}{x_1} = 41.8003$<br>At time $t = 45$, $\frac{x_6}{x_1} = 21.7933$
**Bottom-up**					
(b)	1	2	At time $t = 10$, $x_1 = 0.034605$<br>At time $t = 45$ $x_1 = 2.02039e^{-005}$	At time $t = 10$, $x_6 = 0.965461$<br>At time $t = 45$, $x_6 = 1.8982$	At time $t = 10$, $\frac{x_6}{x_1} = 27.8995$<br>At time $t = 45$, $\frac{x_6}{x_1} = 93952.1$
(d)	0.1	1.1	At time $t = 10$, $x_1 = 0.034605$<br>At time $t = 45$, $x_1 = 2.02039e^{-005}$	At time $t = 10$, $x_6 = 0.153518$<br>At time $t = 45$, $x_6 = 1.09006$	At time $t = 10$, $\frac{x_6}{x_1} = 4.4363$<br>At time $t = 45$, $\frac{x_6}{x_1} = 53953$
(f)	0.1	2	At time $t = 10$, $x_1 = 0.034605$<br>At time $t = 45$, $x_1 = 2.02039e^{-005}$	At time $t = 10$, $x_6 = 0.154113$<br>At time $t = 45$, $x_6 = 1.85515$	At time $t = 10$, $\frac{x_6}{x_1} = 4.4535$<br>At time $t = 45$, $\frac{x_6}{x_1} = 91821.2$
(h) With Slop 1000	0.1	$\cdots$	At time $t = 10$, $x_1 = 0.034605$<br>At time $t = 45$, $x_1 = 2.02039e^{-005}$	At time $t = 10$, $x_6 = 0.151714$<br>At time $t = 45$, $x_6 = 0.132478$	At time $t = 10$, $\frac{x_6}{x_1} = 4.38418$<br>At time $t = 45$, $\frac{x_6}{x_1} = 95125.8$
(j) With Slop 10	0.1	$\cdots$	At time $t = 10$, $x_1 = 0.034605$<br>At time $t = 45$, $x_1 = 2.02039e^{-005}$	At time $t = 10$, $x_6 = 0.151714$<br>At time $t = 45$, $x_6 = 2.10546$	At time $t = 10$, $\frac{x_6}{x_1} = 4.38418$<br>At time $t = 45$, $\frac{x_6}{x_1} = 104211$

Table S5: **Simulating Responses of NH_4^+−Sink Systems to External Step Inputs**

Top-Down					
**Plot No.**	**Step Input (Red)**		**Output**		
	**Upper Level**	**Lower Level**	x_1 **(Black)**	x_6 **(Cyan)**	$\frac{x_6}{x_1}$
(a)	2	1	At time $t = 10$, $x_1 = 0.449569$	At time $t = 10$, $x_6 = 3.52216$	At time $t = 10$, $\frac{x_6}{x_1} = 7.83453$
			At time $t = 45$ $x_1 = 0.495715$	At time $t = 45$, $x_6 = 12.2157$	At time $t = 45$, $\frac{x_6}{x_1} = 24.6425$
(c)	1.1	0.1	At time $t = 10$, $x_1 = 0.449113$	At time $t = 10$, $x_6 = 2.80763$	At time $t = 10$, $\frac{x_6}{x_1} = 6.2515$
			At time $t = 45$, $x_1 = 0.490228$	At time $t = 45$, $x_6 = 4.34883$	At time $t = 45$, $\frac{x_6}{x_1} = 8.87104$
(e)	2	0.1	At time $t = 10$, $x_1 = 0.449569$	At time $t = 10$, $x_6 = 3.52045$	At time $t = 10$, $\frac{x_6}{x_1} = 7.83074$
			At time $t = 45$, $x_1 = 0.492459$	At time $t = 45$, $x_6 = 5.05956$	At time $t = 45$, $\frac{x_6}{x_1} = 10.2741$
(g) With Slop 1000	2	$\cdots$	At time $t = 10$, $x_1 = 0.449026$	At time $t = 10$, $x_6 = 2.69055$	At time $t = 10$, $\frac{x_6}{x_1} = 5.99198$
			At time $t = 45$, $x_1 = 0.494052$	At time $t = 45$, $x_6 = 11.3852$	At time $t = 45$, $\frac{x_6}{x_1} = 23.0445$
(i) With Slop 10	2	$\cdots$	At time $t = 10$, $x_1 = 0.449026$	At time $t = 10$, $x_6 = 2.69055$	At time $t = 10$, $\frac{x_6}{x_1} = 5.99198$
			At time $t = 45$, $x_1 = 0.494052$	At time $t = 45$, $x_6 = 11.3852$	At time $t = 45$, $\frac{x_6}{x_1} = 23.0445$
**Bottom-UP**					
(b)	1	2	At time $t = 10$, $x_1 = 0.449026$	At time $t = 10$, $x_6 = 2.69125$	At time $t = 10$, $\frac{x_6}{x_1} = 5.99352$
			At time $t = 45$ $x_1 = 0.494898$	At time $t = 45$, $x_6 = 14.3014$	At time $t = 45$, $\frac{x_6}{x_1} = 28.8976$
(d)	0.1	1.1	At time $t = 10$, $x_1 = 0.446583$	At time $t = 10$, $x_6 = 0.652029$	At time $t = 10$, $\frac{x_6}{x_1} = 1.46004$
			At time $t = 45$, $x_1 = 0.48643$	At time $t = 45$, $x_6 = 9.7679$	At time $t = 45$, $\frac{x_6}{x_1} = 20.0808$
(f)	0.1	2	At time $t = 10$, $x_1 = 0.446583$	At time $t = 10$, $x_6 = 0.652624$	At time $t = 10$, $\frac{x_6}{x_1} = 1.46137$
			At time $t = 45$, $x_1 = 0.487786$	At time $t = 45$, $x_6 = 12.2667$	At time $t = 45$, $\frac{x_6}{x_1} = 25.1476$
(h) With Slop 1000	0.1	$\cdots$	At time $t = 10$, $x_1 = 0.449026$	At time $t = 10$, $x_6 = 2.69055$	At time $t = 10$, $\frac{x_6}{x_1} = 5.99198$
			At time $t = 45$, $x_1 = 0.494052$	At time $t = 45$, $x_6 = 11.3852$	At time $t = 45$, $\frac{x_6}{x_1} = 23.0445$
(j) With Slop 10	0.1	$\cdots$	At time $t = 10$, $x_1 = 0.449026$	At time $t = 10$, $x_6 = 2.69055$	At time $t = 10$, $\frac{x_6}{x_1} = 5.99198$
			At time $t = 45$, $x_1 = 0.494052$	At time $t = 45$, $x_6 = 11.3852$	At time $t = 45$, $\frac{x_6}{x_1} = 23.0445$

References

[1] Smith, H. Monotone dynamical systems: an introduction to the theory of competitive and cooperative systems. Mathematical surveys and monographs, American Mathematical Society 41 (1995).

[2] Hirsch, M., & Smith, H.L. Monotone dynamical systems. In: Handbook of differential equations, ordinary differential equations (second volume), Elsevier, Amsterdam (2005).

[3] Sontag, E.D. Monotone and near-monotone biochemical networks. Systems and Synthetic Biology 1, 59-87 (2007).

[4] Angeli, D., Sontag, E.D. Multi-stability in monotone input/output systems. System Control Letter 51:185-202 (2004).

[5] Hecke K. Van, Cleemput O. Van , Baert L., Theoretical aspects on chemo-denitrification of nitrate in groundwater. International Symposium, France, November, 1990.

[6] Thomas R., On the relation between the logical structure of systems and their ability to generate multiple steady states or sustained oscillations. Springer Ser Synerget 9:180âĂŞ193 (1981).
